# Immunogenomic Analyses of the Prognostic Predictive Model for Patients With Renal Cancer

**DOI:** 10.3389/fimmu.2021.762120

**Published:** 2021-10-12

**Authors:** Tao Feng, Jiahui Zhao, Dechao Wei, Pengju Guo, Xiaobing Yang, Qiankun Li, Zhou Fang, Ziheng Wei, Mingchuan Li, Yongguang Jiang, Yong Luo

**Affiliations:** ^1^ Department of Urology, Beijing Anzhen Hospital, Capital Medical University, Beijing, China; ^2^ Department of Urology, Beijing Huairou Hospital, Beijing, China; ^3^ Department of Cardiovascular Surgery, Beijing Anzhen Hospital, Capital Medical University, Beijing, China

**Keywords:** renal cell carcinoma, tumor immune microenvironment, prognostic model, risk score, immunotherapy

## Abstract

**Background:**

Renal cell carcinoma (RCC) is associated with poor prognostic outcomes. The current stratifying system does not predict prognostic outcomes and therapeutic benefits precisely for RCC patients. Here, we aim to construct an immune prognostic predictive model to assist clinician to predict RCC prognosis.

**Methods:**

Herein, an immune prognostic signature was developed, and its predictive ability was confirmed in the kidney renal clear cell carcinoma (KIRC) cohorts based on The Cancer Genome Atlas (TCGA) dataset. Several immunogenomic analyses were conducted to investigate the correlations between immune risk scores and immune cell infiltrations, immune checkpoints, cancer genotypes, tumor mutational burden, and responses to chemotherapy and immunotherapy.

**Results:**

The immune prognostic signature contained 14 immune-associated genes and was found to be an independent prognostic factor for KIRC. Furthermore, the immune risk score was established as a novel marker for predicting the overall survival outcomes for RCC. The risk score was correlated with some significant immunophenotypic factors, including T cell infiltration, antitumor immunity, antitumor response, oncogenic pathways, and immunotherapeutic and chemotherapeutic response.

**Conclusions:**

The immune prognostic, predictive model can be effectively and efficiently used in the prediction of survival outcomes and immunotherapeutic responses of RCC patients.

## Background

The prevalence of renal cell carcinoma (RCC), a lethal urogenital cancer, ranks third after prostate and bladder cancers (1–3). In 2020, about 73,750 new RCC cases were diagnosed, with approximately 14,830 deaths in the USA (3). Nowadays, a range of treatments, such as surgery accompanied with or without postoperative adjuvant therapy, chemotherapy, immunotherapy, and target therapy, have been developed for RCC. Although these options have certain therapeutic effects, the overall prognosis of RCC patients remains dismal, especially in the late-stage RCC (4).

Over recent decades, the development of immunotherapy has revolutionized cancer treatment paradigms and has been recognized as a promising therapeutic frontier (5–7). For example, immune checkpoint blockade (ICB) is a new therapeutic strategy for several cancer types, such as breast cancer (8, 9), melanoma (10, 11), and lung cancer (12, 13). ICB has also evolved in RCC and showed certain practical application value through the years based on the phase III CheckMate 025 study, whether or not patients have been previously treated (14, 15). In addition, accumulating evidence has also proven that the tumor immune microenvironment (TIME), which encompasses immune cells, fibroblasts, extracellular matrix, endothelial cells, and various cytokines, is associated with tumor progression and metastasis (16–19). In 2017, Chevrier et al. depicted an in-depth Immune Atlas of Clear Cell Renal Cell Carcinoma by applying mass cytometry for the high-dimensional single-cell analysis of kidney primary Tumors (20). In addition, an increased number of studies have proved that multiple immune cells, including CD8^+^ T cells, CD4^+^ T cells and NK cells et al, have been associated with ccRCC tumor (21, 22). An in-depth understanding of TIME is critical to identifying potential immunotherapeutic targets for RCC. However, the majority of the studies have only evaluated gene expressions in the prediction of survival rates for RCC patients, and most of these biomarkers only reveal the status of TIME in some aspects (23, 24). Hence, a comprehensive immune-based model might provide an in-depth insight into the association between prognosis and TIME in RCC.

In the current study, we established an immune prognostic signature model for RCC using the training cohort and further confirmed the effectiveness of the prognosis model in the testing and the entire cohort. Additionally, the associations between the risk score subtypes and immune checkpoints, antitumor immunity, antitumor response, oncogenic pathways, immune cell infiltration, and tumor mutation burden (TMB) were explored. Also, the models’ ability for the prediction of chemotherapeutic and immunotherapeutic responses was evaluated. Finally, we screened out two compounds that could improve the prognosis of RCC.

## Materials and Methods

### Data Acquisition as Well as Preprocessing

Transcriptional expression profiles, mutation patterns, and related clinical data for KIRC patients were retrieved from the Cancer Genome Atlas (TCGA) cohort (https://cancergenome.nih.gov/). Immune-associated genes (IRGs) were derived from the Immunology Database as well as Analysis Portal (ImmPort) database (25). The immunophenoscore (IPS) for RCC patients were retrieved from the cancer immune group atlas (TCIA) (https://tcia.at/home). In addition, the advanced urothelial cancer database of administered anti-PD-L1 therapy was downloaded using the R package “IMvigor210CoreBiologies” (version 1.0.0) (26). The malignant melanoma dataset that received anti-PD-1 and antiCTLA4 therapy were obtained from the GSE91061 cohort. All data were subjected to background correction and logarithmic conversion using R software.

### Differentially Expressed Immune-Related Genes (DE-IRGs) and Functional Enrichment Analyses

Differential gene expression analysis between tumor and corresponding normal tissues in KIRC were screened based on the count data for TCGA kidney cancer cohort using the R package “DESeq2” (27), according to the screening criteria (log2|fold change| >2, P-value <0.05). The IRGs involved in oncogenesis were provide by IMMPORT website. Then, DE-IRGs were identified by the intersection between DEGs and IRGs.

The R package “clusterProfiler” was used for Gene Ontology (GO) as well as Kyoto Encyclopedia of Genes and Genomes (KEGG) pathway enrichment analyses of these significant DE-IRGs and their visualization (28). Next, we defined the pathways and terms using false discovery rate (FDR) ≤0.05 as statistically significant.

### Establishment of the Immune-Related Risk Score

Among 538 KIRC with mRNA expression data, 517 patients with the overall survival (OS) data were retained for further analyses. First, 70% of samples were randomly drawn and grouped as training cohort to develop a prognostic risk model, and the other 30% of samples comprised the validation set, which was used in evaluating the model’s predictive ability and robustness in the entire cohort. Then, DE-IRGs were screened out by univariate Cox proportional hazard regression through the “coxph” R-function from the “survival” package (29). Subsequently, the least absolute shrinkage and selection operator (LASSO) Cox regression analysis was carried out to select the prognostic genes using the R package “glmnet” (30). Finally, the immune-associated risk score was calculated using LASSO Cox regression hazard regression − retrieved regression coefficients to multiply expression levels of genes (the risk score = mRNA expression levels of gene a × coefficient a + mRNA expression levels of gene b × coefficient b + ……+ mRNA expression levels of gene n × coefficient n).

In addition, by setting the median of risk score as the cutoff value, the patients were classified into a high-risk group and a low-risk group. To establish the prognostic accuracy of the established model, we used Kaplan–Meier survival curve analysis, concordance (C)-index, log-rank test in addition to time-dependent receiver operating characteristic curves (ROC) and XGBoost algorithm.

### Independent Prognostic Value of the Immune-Associated Prognostic Signature

Multivariate Cox regression analysis with the forward stepwise procedure was performed to investigate if the risk score is an independent prognostic factor. The immune-associated risk score and other clinical variables with P <0.05 were identified as independent prognostic risk factors.

### Establishment and Validation of the Nomogram

To develop a prognostic signature for 1-, 3-, and 5-year survival rates, a nomogram was constructed using the identified independent prognostic variables, such as stage, age, and risk score (31). Moreover, the C-index, calibration curve, decision curve analysis (DCA), and ROC analysis were performed to determine its predictive accuracy and discriminatory capacity (32). The C-index was evaluated using a bootstrap method involving 1000 resamples (33). The C-index values, dependent on the nomogram’s predictive ability, ranged from 0.5 (no discrimination) to 1 (perfect discrimination). The consistency between the predictive survival rate and the actual survival rate in unknown samples was assessed using calibration curves. Additionally, DCA (34) was used to evaluate the clinical utility and the net benefits of the nomogram as it takes both discrimination and calibration into consideration. Finally, the area under the receiver operating characteristic (ROC) curve (AUC) was also determined for each variable to evaluate the discriminative performance of the nomograms.

### Immune Cell Proportion Analyses and Immune Related Features

To explore immune cell abundance in KIRC tissues, CIBERSORT (35) was employed to evaluate the proportions of 22 immune cell types using a deconvolution algorithm by the R package with default parameters. In addition, the ESTIMATE scores (ES), tumor purity (TP), stromal scores (SS), and immune scores (IS) for each KIRC sample were evaluated using the ESTIMATE algorithm (19) of the “estimate” package. The cytolytic activity (CYT) index is a geometric mean of mRNA expression levels of GZMA and PRF1, and was utilized to assess the intratumoral immune cytolytic T-cell activities (36).

### Immunotherapy and Chemotherapeutic Response in Risk Score Subtype

As immune checkpoint molecules are widely explored in the immunotherapeutic studies of multiple cancers, programmed cell death 1 (PDCD1, also referred to as PD-1), CD274 molecule (also referred to as PD-L1), and cytotoxic T-lymphocyte protein 4 (CTLA4) were used to evaluate the associations between risk scores and immunotherapeutic efficacies. The urothelial cancer dataset (IMvigor210) comprising of administered anti-PD-L1 therapy was used to establish the therapeutic benefits between high- and low-risk score subtypes using four treatment categories: progressive disease (PD), stable disease (SD), complete response (CR), and partial response (PR).

IPS is a machine learning-based scoring system applied for the prediction of patients’ responses to immune checkpoint inhibitor (ICI) treatment based on the weight average Z scores representing immune-related genes expression in cell types (37). High IPS scores reflect increased immunogenicity.

As chemotherapy and targeted therapy are widely used to treat clear cell renal cell carcinoma (ccRCC), risk scores were used to predict the drug sensitivity based on half-maximal inhibitory concentrations (IC50) for each KIRC patient from the Genomics of Drug Sensitivity in Cancer (GDSC) website (38) using the R package “pRRophetic” (39–44). The common target drugs, such as Cisplatin, Gefitinib, Gemcitabine, Sorafenib, Sunitinib, Vinblastine, Vinorelbine, and Vorinostat, were selected for ccRCC.

### Tumor Mutational Burden (TMB), Connectivity Map (CMAP) and Molecular Docking Analysis

KIRC patients’ somatic variants data were analyzed and visualized by “maftool” R package (45) to identify the mutation burden of KIRC in the high- and low-risk scores. Then, the TMB of each patient was calculated as follows: mutations/million bases.

Next, to identify the potentially small molecules related to this signature, genes in the model were assessed *via* CMAP analysis. Thus, the positive mean represented that these selected drugs may share similar functions with the model, while the negative mean indicated that these drugs could improve the prognosis of RCC. Herein, we screened compounds by setting the criteria as P <0.05.

Moreover, the crystal structure of the protein was obtained from RCSB Protein Data Bank. The three-dimensional structures for all compounds were downloaded from PubChem database using MOL2 format. The molecular docking calculations were conducted using Schrodinger and Pymol 2.1 software.

### Statistical Analysis

The differences between variables were determined by chi-square as well as Student’s t-tests. For baseline clinical data, the Wilcoxon test and the Kruskal–Wallis were utilized to evaluate the significant differences between two or multiple groups, respectively. The Kaplan–Meier survival curves were compared using the log-rank test. P<0.05 indicated statistical significance. R 4.0.3 and SPSS 26.0 software were used for all analyses.

## Results

### Identification and Functional Analyses of DE-IRGs

All 517 KIRC samples with OS information were split into training (367 patients) and test groups (151 patients). Between the training and validation cohorts, no significant differences were detected among most of the clinical characteristics (**Table 1**).

**Table 1:** The clinical characteristics of KIRC patients.

Variables	Group	Total set (n = 517)	Training set (n = 367)	Testing set (n = 151)	P value
**Vital status**	**Alive**	361	249	111	0.136
	**Dead**	156	118	38	
**Survival time**		1054.797	1046.125	1070.073	0.815
**Clinical Stage**	**I**	257	189	68	0.233
	**II**	54	36	19	
	**III**	123	80	43	
	**IV**	83	62	21	
**T stage**	**T1**	262	192	70	0.637
	**T2**	65	46	20	
	**T3**	179	122	57	
	**T4**	11	7	4	
**N stage**	**N0**	233	166	67	0.348
	**N1**	15	8	7	
	**NX**	269	193	77	
**M stage**	**M0**	413	286	128	0.144
	**M1**	78	59	19	
	**MX**	26	22	4	
**Grade**	**G1**	13	11	2	0.478
	**G2**	225	161	65	
	**G3**	204	146	58	
	**G4**	75	49	26	
**Gender**	**Male**	339	236	104	0.32
	**Female**	178	131	47	
**Age**	**<65**	326	233	94	0.791
	**≥65**	191	134	57	

**Figure 1:**
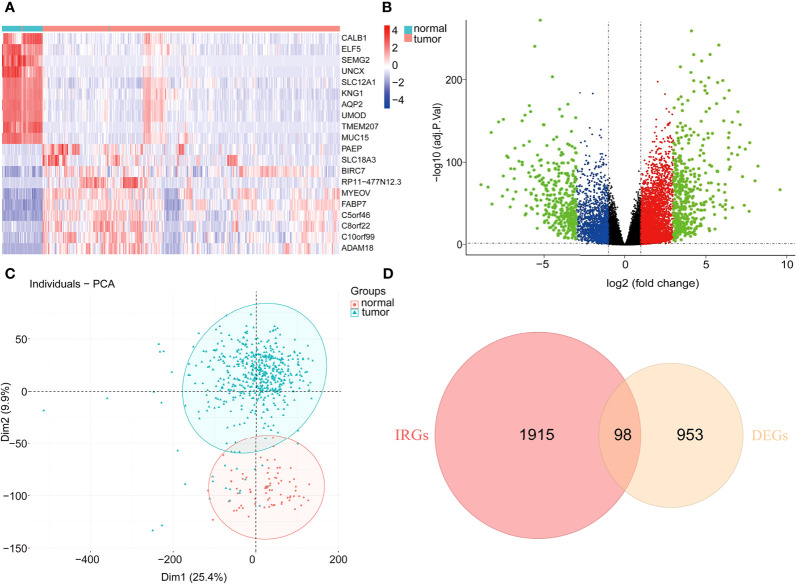
Differentially expressed immune-associated genes. **(A)** Heatmap of top 10 up- and down-regulated genes between normal and tumor tissues. **(B)** Volcano plot for DEGs between normal and tumor tissues. **(C)** PCA plot of the data. **(D)** Venn diagram for intersections between DEGs and IRGs.

With the cutoff value |log2 fold change (logFC)|>2 and adjusted P<0.05, 953 DEGs were filtered, of which 539 genes were significantly elevated, while 414 genes were significantly suppressed in tumor samples compared to normal samples (**Figures 1A, B** **)**. Moreover, principal component analysis (PCA) results **(** **Figure 1C** **)** revealed that KIRC samples clustered separately from normal samples. Subsequently, the intersection between DEGs and immune-associated genes retrieved from the ImmPort database was determined, and 98 DE-IRGs were selected and visualized on a Venn diagram **(** **Figure 1D** **)**.

**Figure 2:**
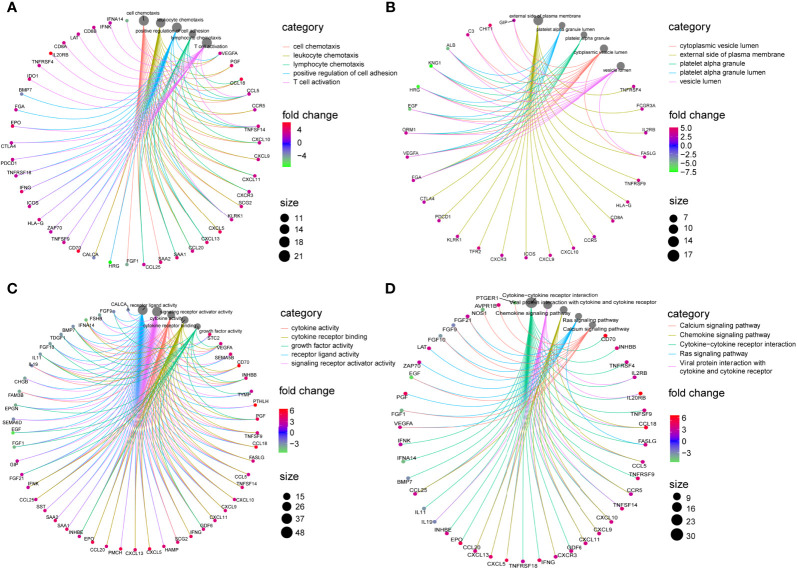
Enrichment analysis of DE-IRGs. **(A)** Visualization of top 5 enriched GO analysis in BP. **(B)** Visualization of top 5 enriched GO analysis in CC. **(C)** Visualization of top 5 enriched GO analysis in MF. **(D)** Visualization of top 5 enriched KEGG pathways.

These 98 DE-IRGs were further utilized in functional enrichment analyses, including KEGG and GO analyses. Based on GO analysis, in the biological process (BP), these DE-IRGs were enriched in cell chemotaxis, leukocyte chemotaxis, lymphocyte chemotaxis, positive regulation of cell adhesion, and T cell activation (**Figure 2A**). In the cellular component (CC) category, the DE-IRGs were mainly enriched in the cytoplasmic vesicle lumen, plasma membrane’s external side, platelet alpha granule, platelet alpha granule lumen, and vesicle lumen (**Figure 2B**). Regarding molecular function (MF), these DE-IRGs were enriched in cytokine receptor binding, growth factor activity, receptor ligand activity, cytokine activity, and signaling receptor activator activity (**Figure 2C**). Regarding KEGG pathways analysis, these DE-IRGs were mainly involved in the calcium signaling pathway, chemokine signaling pathways, cytokine-cytokine receptor interactions, Ras signaling pathways, and viral protein interactions with cytokine receptors and cytokines (**Figure 2D**).

### Establishment and Validation of Prognostic Immune Score Model

**Figure 3:**
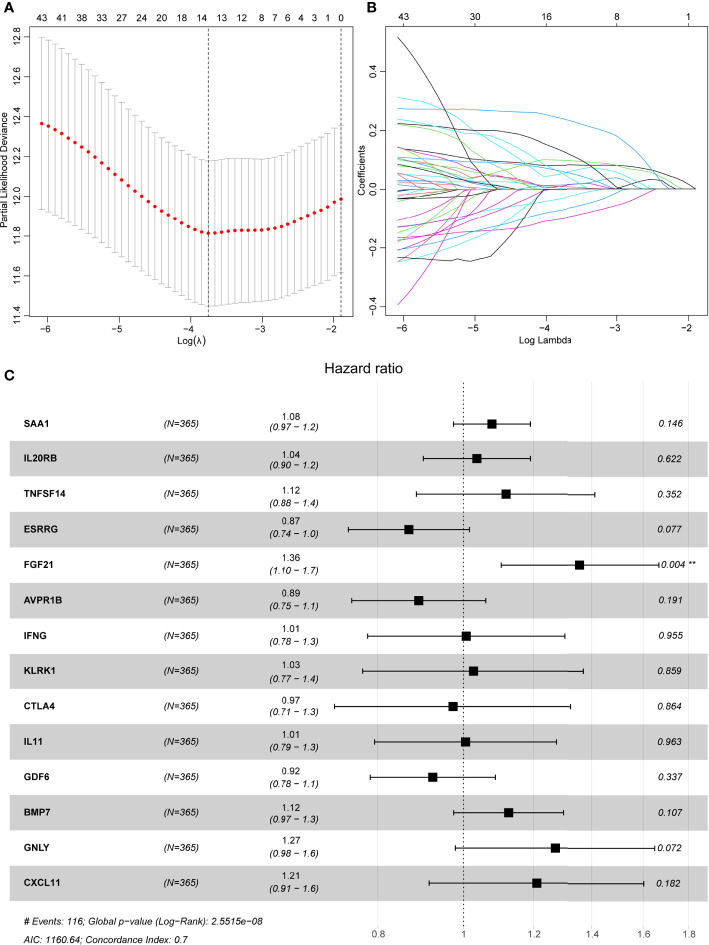
LASSO regression analyses and a forest plot describing Cox regression model findings of 14 immune-associated genes. **(A)** Partial likelihood deviance with changing of log (λ) plotted by LASSO regression in 10-fold cross-validations. Vertical dotted lines were described at the optimal values using minimum criteria and the 1-SE criteria. **(B)** The LASSO coefficient profiles for 14 DE-IRGs in the 10-fold cross-validation. **(C)** Forest plot representing correlations between the expression levels of 14 DE-IRGs and overall survival outcomes in the training dataset. HR, 95% CI, and P-values were evaluated by LASSO regression analyses. ** means P < 0.01.

All 367 KIRC samples in the training cohort were utilized in a prognostic model establishment. First, univariate Cox regression analysis was carried out to explore the association between DE-IRGs and the OS outcomes for KIRC samples. Among 98 DE-IRGs, 47 genes were selected. To avoid overfitting, we further conducted the LASSO Cox regression analysis with minimized lambda (**Figures 3A, B** **)**. A total of 14/47 genes were used to establish the prognostic immune score model using the following formula: risk score = (SAA1 × 0.08215) + (IL20RB × 0.07643) + (TNFSF14 × 0.09743) + (ESRRG × -0.09743) + (FGF21 × 0.23324) + (IFNG × 0.05956) + (CTLA4 × 0.01439) + (KLRK1 × 0.00717) + (IL11 × 0.01639) + (GDF6 × - 0.02484) + (BMP7 × 0.05433) + (GNLY × 0.11780) + (AVPR1B × -0.06460) + (CXCL11 × 0.03907) (**Figure 3C**).

**Figure 4:**
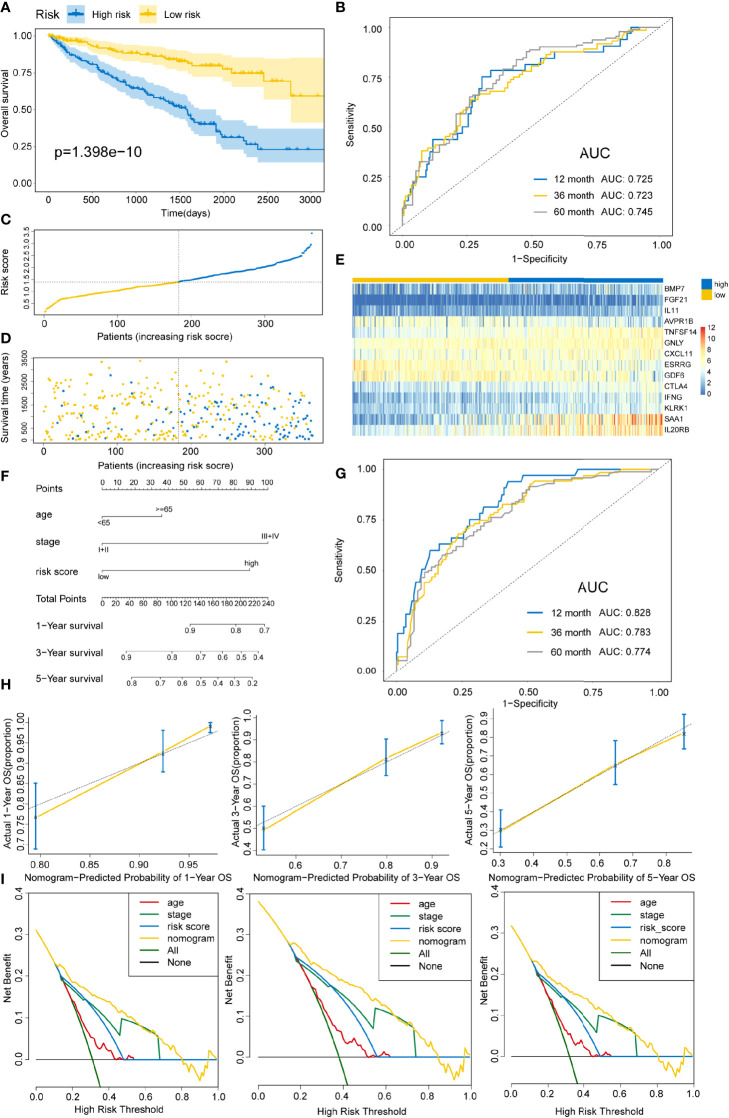
Constructing an immune risk score predictive model using the training set. **(A)** Kaplan–Meier curves for OS outcomes in the training cohort grouped into high- and low-risk score groups. **(B)** Time-dependent ROC curves for prediction of 1-, 3-, and 5-year survival outcomes. **(C)** Distribution of risk scores of the training cohort. **(D)** Vital statuses for patients in high- and low-risk patients. **(E)** Expression patterns for 14 immune-associated genes in high- and low-risk score cohorts. **(F)** A nomogram for the estimation of 1-, 3-, and 5-year OS probabilities in the training cohort. Risk scores and other independent prognostic factors are incorporated in the model. **(G)** Time-dependent ROC curves for the prediction of 1-, 3-, and 5-year survival rates using the nomogram. **(H)** Calibration plot of nomogram in the training cohort according to the agreement between predicted and observed 1-, 3-, and 5-year outcomes. The model’s ideal performance is shown by dashed lines. **(I)** Decision curve analysis for 1-, 3-, and 5-year risk using the nomogram. Black line represents the hypothesis that no patient died at 1-, 3-, and 5-years.

Based on the above calculated formula, the risk scores for every patient in the training set were computed. Then, with the median risk score as the basis, patients were allocated into the high- and low-risk groups. The high-risk patients exhibited significantly poor OS outcomes compared to low-risk patients (P=1.398E-10) (**Figure 4A**). As illustrated in **Figure 4B**, the AUCs of risk scores for 1-, 3-, and 5-years were 0.725, 0.723, and 0.745, respectively, in the training group. The distribution of the risk scores, survival time, survival status, and the expression of 14 OS-associated DE-IRGs for KIRC patients in the training cohort are displayed in **Figures 4C–E**. The model’s C-index was 0.698 (95% confidence interval (CI): 0.647–0.750, P=6.849E-14). To further explore whether the prognostic model was independent of other clinical elements, such as grade, age, T stage, and clinical stage, univariate and multivariate Cox regression analyses were conducted **(** **Table 2** **)**. The risk score was confirmed as an independent prognostic factor (HR=2.699, 95% CI: 1.716–4.243, P<0.0001).

**Table 2:** Univariate and multivariate Cox regression analysis.

Variables	Univariate analysis		Multivariate analysis	
	HR (95%CI)	P value	HR (95%CI)	P value
Training set				
Age (≥65 vs <65)	1.595 (1.106~2.301)	0.012	1.518 (1.044~2.29)	0.0291
Grade (G3+4 vs G1+2)	2.427 (1.601~3.679)	<0.001	1.264 (0.803~1.990)	0.3109
T stage (T3+4 vs T1+2)	3.108 (2.143~4.508)	<0.001	1.986 (1.028~3.915)	0.0514
Stage (III+IV vs I+II)	4.066 (2.749~6.015)	<0.001	5.384 (2.551~11.369)	<0.001
Risk score (high vs low)	3.678 (2.372~5.703)	<0.001	2.699 (1.716~4.243)	<0.001
Testing set				
Age (≥65 vs <65)	2.015 (1.061~3.826)	0.032	2.737 (1.412~5.305)	0.003
Grade (G3+4 vs G1+2)	5.956 (2.311~15.350)	<0.001	2.318 (0.788~6.821)	0.127
T stage (T3+4 vs T1+2)	5.279 (2.543~10.957)	<0.001	2.443 (0.321~18.575)	0.127
Stage (III+IV vs I+II)	5.265 (2.480~1.177)	<0.001	1.510 (0.199~11.461)	0.69
Risk score (high vs low)	10.371 (3.656~29.418)	<0.001	7.991 (2.684~23.786)	<0.001
Total set				
Age (≥65 vs <65)	1.674 (1.220~2.298)	0.001	1.734 (1.262~2.383)	0.001
Grade (G3+4 vs G1+2)	2.887 (1.987~4.196)	<0.001	1.766 (1.189~2.622)	0.005
T stage (T3+4 vs T1+2)	3.468 (2.500~4.811	<0.001	1.352 (0.722~2.530	0.346
Stage (III+IV vs I+II)	4.248 (3.012~5.993)	<0.001	3.903 (2.018~7.549)	<0.001
Risk score (high vs low)	3.358 (2.316~4.868)	<0.001	2.424 (1.648~3.563)	<0.001

In addition, a quantitative strategy for the prediction of the prognostic outcomes of patients was established by constructing a nomogram that integrated the risk scores as well as other independent clinical prognostic factors for OS (**Figure 4F**). Then, the nomogram’s performance was determined using the ROC curve, C-index, calibration curve, and decision curve analyses. The AUCs of the nomogram were 0.828, 0.783, and 0.774 for 1-, 3-, and 5-year survival times, respectively (**Figure 4G**). The C-index was 0.762 (95% CI: 0.720–0.804, P=1.800E-34). Based on the calibration curve, the training cohort predicted that 1-, 3-, and 5-year survival probabilities were good (**Figure 4H**). For the decision curve, the nomogram exhibited a higher net benefit than other schemes to predict the OS (**Figure 4I**).

**Figure 5:**
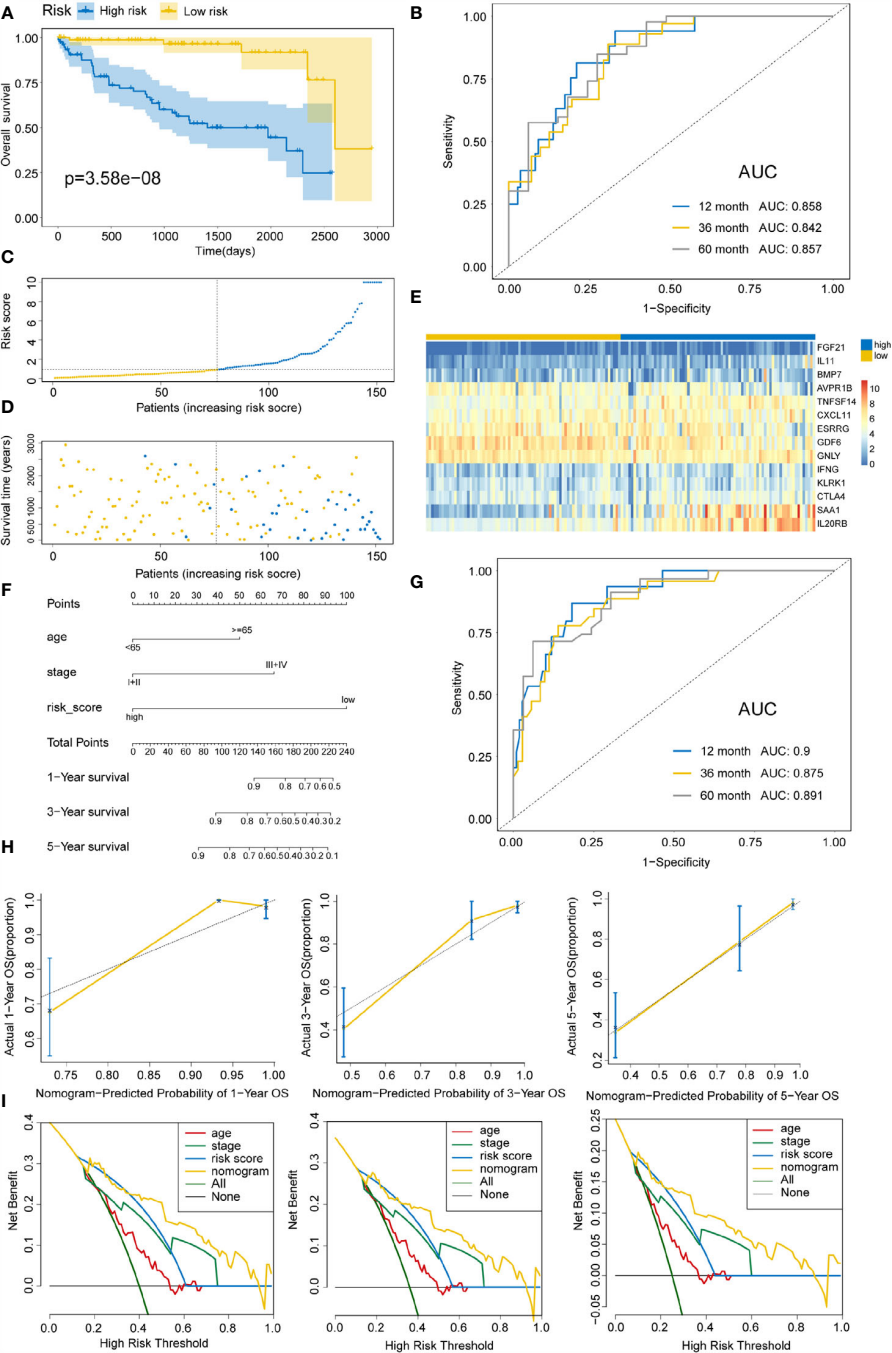
Validating immune risk score prognostic predictive model in the testing set. **(A)** Kaplan–Meier curves for OS outcomes in the testing cohort divided by high- and low-risk score groups. **(B)** The time-dependent ROC curves for predicting 1-, 3-, and 5-year survival outcomes using this signature. **(C)** Risk score distribution in the testing cohort. **(D)** Vital statuses of patients in high- and low-risk patients. **(E)** Expression patterns for 14 immune-associated genes in the high- and low-risk score cohorts. **(F)** Nomogram developed for the prediction of probabilities for 1-, 3-, and 5-year OS outcomes in the testing cohort. Risk scores and other independent prognostic factors were incorporated in the nomogram. **(G)** Time-dependent ROC curves for prediction of 1-, 3-, and 5-year survival outcomes using the nomogram. **(H)** Calibration plot of nomogram in the training cohort according to the agreement between estimated and observed 1-, 3-, and 5-year outcomes. Dashed lines represent the nomograms’ ideal performance. **(I)** Decision curve analysis for 1-, 3-, and 5-year risk using the nomogram. Black line represents the hypothesis that no patient died after 1-, 3-, and 5-years.

**Figure 6:**
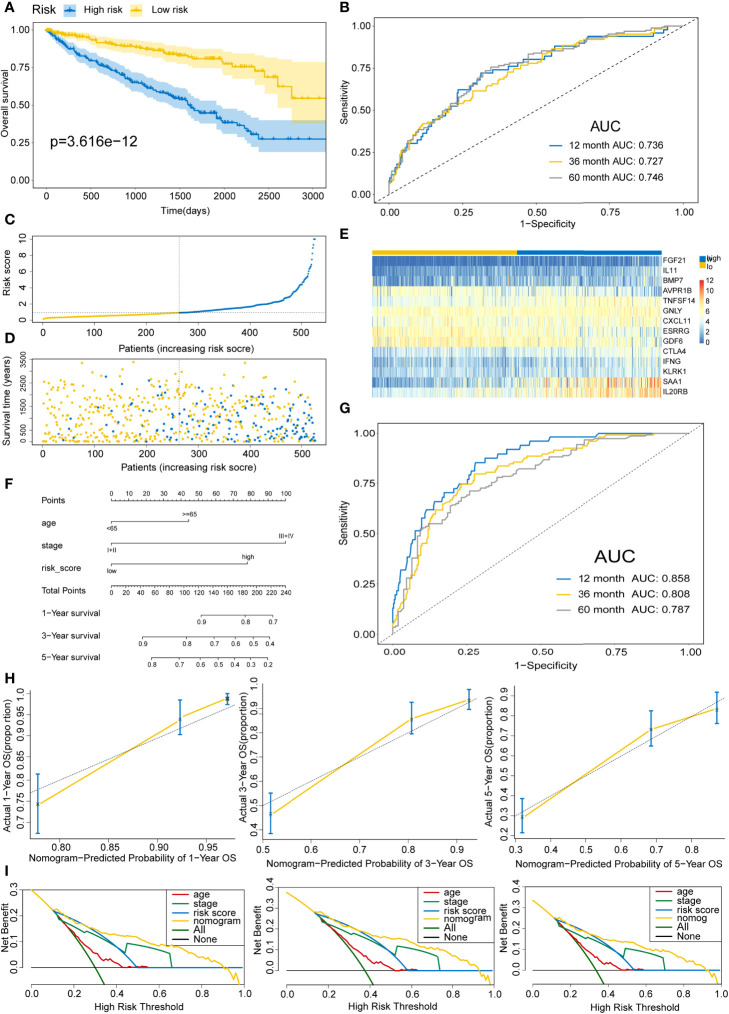
Validating the immune risk score prognostic predictive model for the entire set. **(A)** Kaplan–Meier curves of the OS outcomes in the entire cohort divided as high- and low-risk score groups. **(B)** Time-dependent ROC curves for prediction of 1-, 3-, and 5-year survival outcomes using this signature. **(C)** Risk score distributions for the entire cohort. **(D)** Vital statuses for high- and low-risk group patients. **(E)** Expression patterns for 14 immune-associated genes in the high- and low-risk score cohorts. **(F)** Nomogram for the prediction of the probability of 1-, 3-, and 5-year OS outcomes in the entire cohort. Risk scores and other independent prognostic factors were incorporated into the model. **(G)** Time-dependent ROC curves for prediction of 1-, 3-, and 5-year survival outcomes using the nomogram. **(H)** Calibration plot of nomogram in the training cohort according to the agreement between observed and predicted 1-, 3-, and 5-year outcomes. The models’ ideal performance is shown by the dashed lines. **(I)** Decision curve analysis for 1-, 3-, and 5-year risks using the nomogram. Black line represents the hypothesis that no patient died after 1-, 3-, and 5-years.

To delineate the robustness and versatility of the immune score model, the risk score in the training cohort was validated in the testing and entire cohorts. The participants in the testing and entire cohorts were grouped into high- and low-risk score subtypes using the same formula. The findings in the testing and entire datasets were similar. The Kaplan–Meier survival curves revealed poor survival rates for the high-risk group in the testing (P=3.58E-8) (**Figure 5A**) and the entire cohorts (P=3.616E-12) (**Figure 6A**). The AUC for 1-, 3-, and 5-years are 0.858, 0.842, and 0.857 in the testing group (**Figure 5B**) and 0.736, 0.727, and 0.746 in the entire group (**Figure 6B**). The survival data, risk score, scatterplots, and gene expression pattern distributions in the testing and entire cohorts are shown in **Figure 5C–E** and **Figures 6C–E**. The C-indices of the model were 0.835 (95% CI: 0.782–0.888, P=1.034E-35) and 0.709 (95% CI: 0.666-0.752, P=7.520E-22) in the testing and entire cohorts, respectively. Univariate and multivariate Cox regression analyses for clinicopathological parameters were carried out in the testing and entire cohorts. Also, the risk score was an independent prognostic indicator of OS in KIRC patients (**Table 2**). To improve the prognostic immune score model, the nomogram system was established based on testing and entire cohorts (**Figure 5F** and **Figure 6F**). The AUC of our nomogram for predicting 1-, 3-, and 5-year OS was 0.9, 0.875, and 0.891, respectively, in the testing cohort and 0.858, 0.808, and 0.787, respectively, in the entire cohort (**Figure 5G** and **Figure 6G**). The C-indices of the nomogram in the testing and entire cohorts were 0.859 (95% CI: 0.809–0.909, P=4.980E-45) and 0.786 (95% CI: 0.752-0.821, P=4.201E-59), respectively. Finally, the calibration curves and decision curves for 1-, 3-, and 5-year survival probabilities were established (**Figures 5H, I** and **Figures 6H, I** **)**. These findings indicated that the nomogram has excellent predictive performance in all cohorts.

### Associations Between DE-IRGs Signature and Clinical Characteristics of KIRC Patients

Next, we further investigate the association between clinical characteristics, including tumor burden, age at diagnosis, gender, grade, clinical stage, T stage, and the prognostic risk signature. A significant correlation was established between high-risk score and a high tumor burden (P=9.87E-08), male gender (P=0.03), advanced grade (P=2.54E-09), higher stage (P=8.85E-11), and T stage (P=5.6E-08) (**Figure S1**). Additionally, no statistical significance was observed between < 65-year-old group and >65-year-old group (P=0.1). Subsequently, we also assessed whether the model could assess the survival probability in subgroups exhibiting varying clinical patterns. The prognostic model could be utilized for the prediction of survival probabilities for various clinicopathological parameters (P<0.05) (**Figure S2**).

### Immune Cell Proportions Between High- and Low-Risk Score Patients

**Figure 7:**
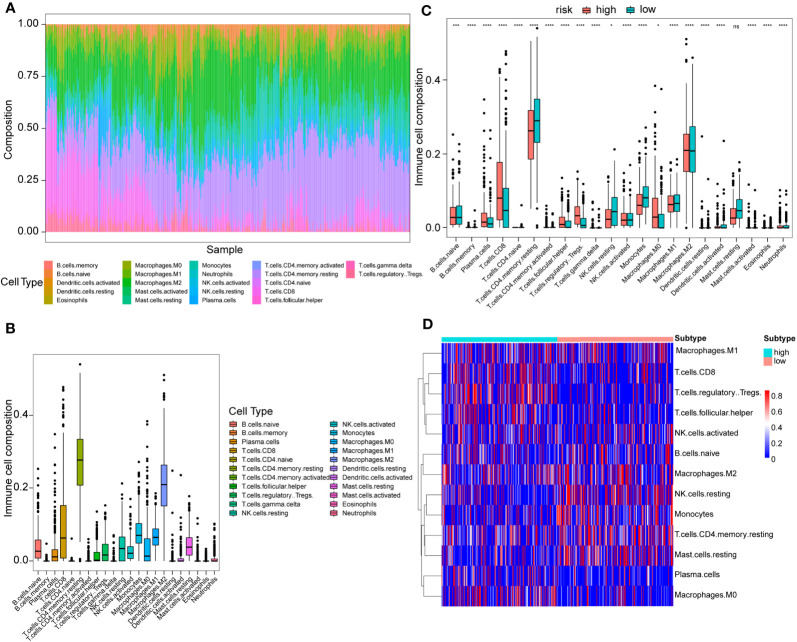
Immune cell proportion analyses in the TCGA cohort between high- and low-risk score patients. **(A)** Overall view of relative proportions of immune cell infiltrations for 22 immune signatures. **(B)** Boxplots for 22 immune cell proportions in the TCGA cohort. **(C)** Boxplots for different immune cell infiltrations in the high- and low-risk score patients. Significance: ns≥0.05, ^∗^<0.05, ^∗∗∗^<0.001, and ^∗∗∗∗^<0.0001. **(D)** Immune cell heatmap for patients in the high- and low-risk score subtypes. Only immune cells whose non-zero proportions exceeded half in all samples were plotted.

Using the CIBERSORT algorithm, 22 immune cell types were determined in each KIRC sample between high- and low-risk score subtypes. The proportions of 22 immune cells and their distribution in tumor samples are illustrated in **Figure 7A** and **Figures 7B, C**, respectively. Compared to the low-risk group, the high-risk score group exhibited significantly elevated proportions of plasma cells, T cells CD8^+^, T cells follicular helper, T regulatory cells (Tregs), and M0 macrophages (P<0.05) (**Figures 7C, D** **)**. Conversely, the proportions of macrophages M1, activated natural killer (NK) cells, naïve B cells, macrophages M2, resting NK cells, monocytes, T cells CD4^+^ memory resting, and resting mast cells in the high-risk score subtype were remarkably elevated compared to those in the low-risk score subtypes (P<0.05) (**Figures 7C, D** **)**. In addition, in 22 immune cell types, high plasma cells, Tregs, follicular helper T cells, and monocytes M0 level were remarkably correlated with poor OS outcomes (P=0.01, 0.0019, <0.0001, and 0.031, respectively), while the increase in activated dendritic cells was related to better OS (P=0.0079) (**Figure S3**). **Figure S4** displayed a weak or moderate correlation between the levels of various tumor-infiltrating immune cells and the risk score.

### Immune Landscape in KIRC Patients

**Figure 8:**
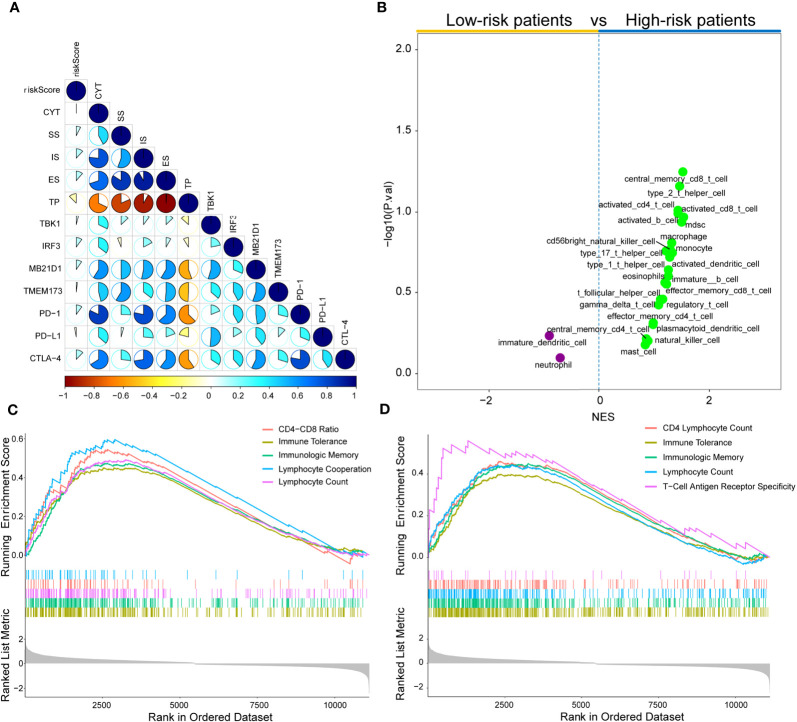
Immune landscape of risk score in the TCGA cohort. **(A)** Correlations between risk score, levels of expression of PD-L1, CYT, TBK1, IRF3, MB21D1, CTLA-4, PD-1, and TMEM173, immune score, stromal score, ESTINATE score, and tumor purity in the TCGA cohort. **(B)** Volcano plots for immune cell sub-population enrichment in high- and low-risk patients according to NES scores from ssGSEA. **(C)** Gene set enrichment analyses described the MeSH terms correlated with risk score using gendoo term in the TCGA cohort. **(D)** Gene set enrichment analysis described the MeSH terms correlated with the risk score using gene2pubmed term in the TCGA cohort.

Subsequently, the associations between risk score and some immune-associated features were assessed. The cGAS-STING pathway has been shown to be a key signaling pathway in antitumor immunity and cancer therapeutics (46–48). Thus, four key genes (*TBK1*, *IRF3*, *MB21D1*, and *TMEM173*) in the cGAS-STING signaling pathway, three immune checkpoint molecules (PD-L1, CTLA-4, and PD-1), CYT, and the results of ESTIMATE algorithm (SS, IS, ES, and TP) and risk score were investigated. **Figure 8A** shows that the risk score values are correlated with the immune score, tumor purity, TBK1, ESTIMATE score, IRF3, stromal score, MB21D1, PD-1, and CTLA-4. **Figure S5** showed significant differences in the CYT, immune score, ESTIMATE score, stromal score, and tumor purity based on the Wilcoxon test between the two risk score subtypes (P<0.0001). Importantly, the expression of IRF3, MB21D1, TMEM173, PD-1, and CTLA-4 was elevated in the high-risk than in the low-risk score subtype.

To further characterize immune cell infiltration, 28 immune cell signatures (25, 49–53) from diverse resources were investigated based on the single sample gene set enrichment analysis (ssGSEA) algorithm. As shown in **Figure 8B**, 23 immune subpopulations (multiple T cell signatures, including T helper cells, central memory CD8+ T cells, and activated CD T cells) were enriched in high-risk patient cohort, whereas only two subpopulations (immature dendritic cells and neutrophils) were enriched in the low-risk patient group. Furthermore, DEGs between low- and high-risk groups were determined by gene set enrichment analysis (GSEA) using two MeSH terms (gene2pubmed and gendoo) to explore their immune-related functions. The DEGs were enriched in multiple immune-associated terms, including CD4-CD8 ratio, immune tolerance, lymphocyte cooperation, lymphocyte count, immunologic memory, and T-cell antigen receptor specificity in gendoo and gene2pubmed (**Figures 8C, D** **)**.

### Correlation Between Risk Score Model and T Cell Infiltrations, Antitumor Immunity, Antitumor Responses, and Oncogenic Pathways

Several studies have shown that cDC1 cells play a central role in the initiation of antitumor CD8^+^ T cells and driving tumor-specific CD^+^8 T cells by activating CXCL10 (54–57). Some studies (57–59) also clarified that the two key chemokines (CCL4 and CCL5) are the key modulators of cDC1 recruitment into tumors *via* activating CCR5 expression. Moreover, chemokines CXCR3, CXCL9, and CXCL10 have been documented on T cell infiltration and NK cell recruitment (60). Thus, we investigated the expression level of CCL4, CXCR3, CXCL9, CCL5, and CXCL10 between high- and low-risk subtypes and the correlations between these genes and the risk score. The high-risk group patients exhibited higher expression levels compared to low-risk patients (P<0.05) (**Figures S6A–E**). Moreover, strong positive correlations were established between risk scores and CXCR3, CCL5, CXCL9, CCL4, and CXCL10 (P<0.05) (**Figures S6F–J**).

Moreover, we explored the association between risk scores, T cell infiltrations, and antitumor response scores (BATF3_DC, IFNA, IFNG, IL_1_speed, T cell_infiltration_1, T cell_infiltration_2, TFNA, and TNFa_speed) determined by ssGSEA from the corresponding TME gene signatures (57, 61). For the high-risk group, the ssGSEA scores for T cell infiltrations and antitumor responses were significantly elevated compared to the low-risk group, as determined by the Wilcoxon test (P<0.05) (**Figure S7A**). A strong positive correlation was established between risk scores and ssGSEA scores save to BATF3_DC (P<0.05) (**Figures S7B–I**). Conclusively, high-risk score patients exhibited elevated T cell infiltration levels.

The differences in the normalized enrichment score (NES) value of 10 oncogenic pathways between low- and high-risk groups were calculated using ssGSEA algorithm; also, the correlation between the NES value and the risk score was evaluated. Compared to the low-risk group, cell cycle and TP53-related pathways exhibited significantly elevated NES values in the high-risk patient group, whereas the Hippo-, NRF2-, PI3K-, RAS-, and TGF-β-related pathways in the high-risk patient group had lower NES value (P<0.05) (**Figure S8A**). The correlations between the risk score and the NES value in the cell cycle (P=1.44e-12) and TP53-related (P=0.024) pathways were found to be positive (**Figure S8B**). Nevertheless, we also observed that the NES value of the Hippo-, NRF2-, PI3K-, RAS-, and TGF-β-related pathways had a negative correlation with the risk score (**Figure S8B**).

### Therapeutic Benefit of the Risk Score

**Figure 9:**
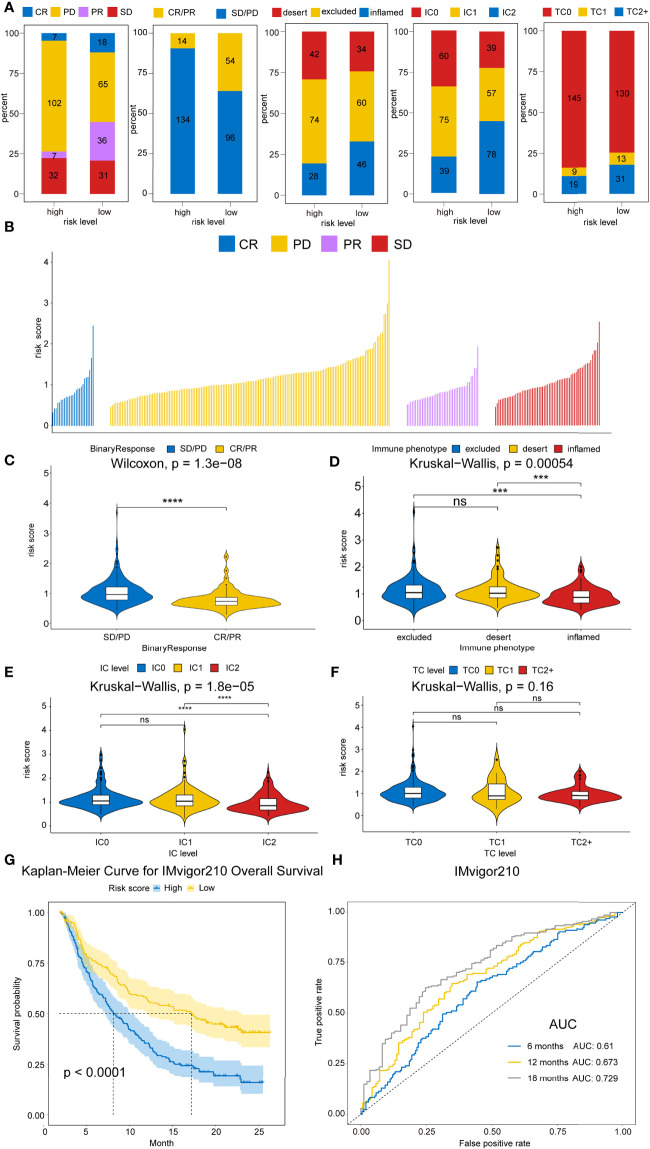
Therapeutic benefits of risk scores calculated by our model. **(A)** Bar graphs illustrate the distribution of the clinicopathological parameters for IMvigor210 dataset in high- and low-risk patients based on chi-square test. (P=4.8008E-08, P=4.8008E-08, P=0.3305, P=6.0E-6, and P=1.6023E-59, respectively). **(B)** Waterfall plot illustrates the risk score distributions for patients exhibiting different immunotherapeutic responses in the IMvigor210 dataset. **(C)** Violin plot illustrates the risk score distributions for patients exhibiting different anti-PD-L1 immunotherapies in IMvigor210 dataset. **(D)** Violin plot illustrates the risk score distributions for patients exhibiting different immune phenotypes in the IMvigor210 dataset. **(E)** Violin plot illustrates the risk score distributions for patients with varying IC levels in the IMvigor210 dataset. **(F)** Violin plot illustrates the risk score distributions for patients with varying TC levels in the IMvigor210 dataset. **(G)** Kaplan–Meier curves for OS outcomes in the IMvigor210 cohort assigned into high- and low-risk score groups. **(H)** Time-dependence ROC curves of anti-PD-L1 immunotherapy response prediction at 0.5-, 1-, and 1.5-year survival rate in the IMvigor210 dataset. Significance: ns≥0.05, ^∗∗∗^<0.001, and ^∗∗∗∗^<0.0001.

Recently, ICB therapies have exhibited striking clinical benefits. However, the main challenge faced by ICB therapies is the limitation of effective predictive markers with only a few patients showing therapeutic response. Herein, the urothelial cancer database (IMvigor210) consisting of anti-PD-L1 therapy and the malignant melanoma database (GSE91016) administered with anti-PD-1 and-CTLA-4 therapy were used to investigate the association between risk score and immunotherapeutic benefits. **Figures 9A–F** and **Figures S9A–C** showed the distribution of clinical and molecular characteristics (immunotherapy response, binary response, immune phenotype, immune cells (IC) level, and tumor cells (TC) level and correlation with risk scores between high- and low-risk groups in the IMvigor210 cohort and GSE91061 cohort separately. For the immunotherapy response, the risk score of RCC with CR/PR were significantly lower than those of RCC with SD/PD, as assessed by the chi-squared test (IMvigor210 dataset: P<0.001, GSE91061 dataset: P=0.036) (**Figure 9A** and **Figure S9C**). The violin plot further revealed that the risk scores in CR/PR were lower than those in SD/PD, as assessed by the Wilcoxon test (IMvigor210 cohort: P=1.3e-08, GSE91061 cohort: P=0.0075) (**Figure 9C** and **Figure S9B**). Strikingly, Kaplan–Meier curves showed that high-risk score patients exhibited worse prognosis compared to the low-risk score patients in IMvigor210 (P<0.0001) (**Figure 9G**) and GSE91061 cohort (P=0.00016) (**Figure S8D**). In addition, IMvigor210 and GSE91061 were used to plot a time-dependent ROC. The current results displayed that the AUCs of our model for OS were 0.61 at 6 months, 0.673 at 12 months, and 0.729 at 18 months in the IMvigor210 cohort (**Figure 9H**) and 0.746 at 12 months, 0.712 at 18 months, and 0.753 at 24 months in the GSE91061 cohort (**Figure S9A**).

**Figure 10:**
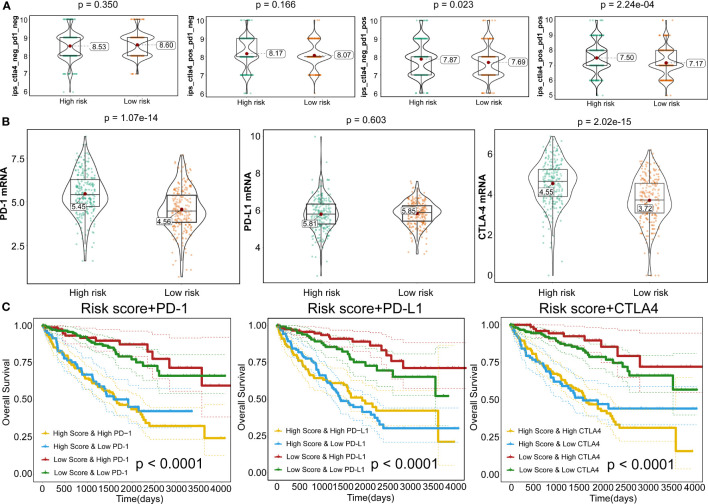
Responses to immune checkpoint inhibitors. **(A)** Violin plots illustrate the relative probabilities for anti-PD-1 and anti-CTLA-4 treatment responses between high- and low-risk groups. **(B)** Violin plots for expression levels of PD-1, CTLA-4, and PD-L1 between high- and low-risk patients. **(C)** Kaplan–Meier curves for OS outcomes among four groups, according to risk score and PD-1, CTLA-4, and PD-L1.

To further expand this study, the machine learning-based score (IPS) was determined to predict patients’ response to ICI treatment. Four subtypes of IPS values (CTLA4_neg_PD1_neg, CTLA4_pos_PD1_neg, CTLA4_neg_PD1_pos, and CTLA4_pos_PD1_pos) were carried out to predict the KIRC patients’ responses to anti-CTLA4 and anti-PD1 treatment. We found that relative probabilities to response to anti-PD1 were elevated in high-risk score patients (P=0.023), and the similar results were obvious in the combination treatment of anti-PD1 and anti-CTLA4 (P=2.24e-04) (**Figure 10A**). In addition, *CTLA-4* and *PD-1* mRNA expression levels in the high-risk score group were significantly elevated compared to the low-risk score patients (P=1.07e-14 and P=2.02e-15), whereas no obvious difference was detected in the *PD-L1* mRNA expression level between high- and low-risk patients (P=0.603) (**Figure 10B**). This phenomenon was consistent with the concept that high expression of ICI genes had a poor prognosis. Owing to the complex environment between immune infiltration and ICI genes, we further examined whether immune infiltration had consequences on the clinical prognosis in ICI genes. **Figure 10C** shows that low-risk score patients with high PD-1 exhibited better clinical outcomes compared to high-risk score and high PD-1, and the outcomes of low-risk score patients with low PD-1 were superior to those of high-risk score patients and low PD-1 levels (P<0.0001). Also, patient groups showed similar findings, and survival patterns were yielded using risk score and PD-L1 or CTLA4 (P<0.0001) (**Figures 10C** **)**.

**Figure 11:**
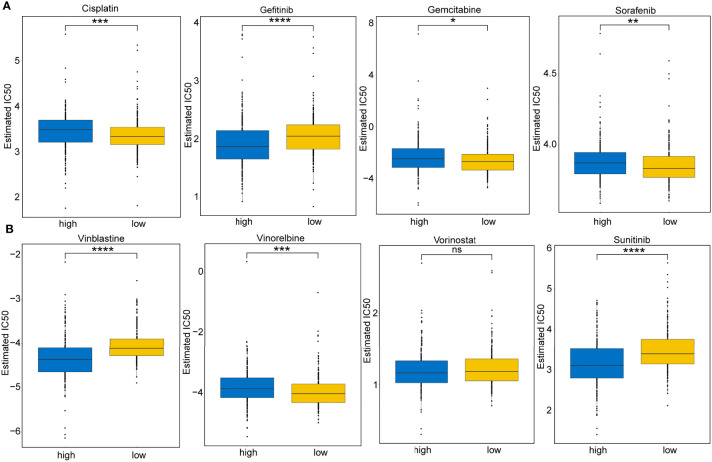
Immunotherapeutic and chemotherapeutic responses for high- and low-risk patients. **(A)** Boxplots illustrate the immunotherapeutic and chemotherapeutic responses of Cisplatin, Gefitinib, Gemcitabine, and Sorafenib in the high- and low-risk patients. **(B)** Boxplots illustrate the immunotherapeutic and chemotherapeutic responses of Vinblastine, Vinorelbine, Vorinostat, and Sunitinib in the high- and low-risk patients. Significance: ns≥0.05, ^∗^<0.05, ^∗∗^<0.01, ^∗∗∗^<0.001, and ^∗∗∗∗^<0.0001.

The responsive predictive values of the risk score to chemotherapy and target-therapy were also investigated by the IC50 of eight drugs. The estimated IC50 values of Cisplatin, Gemcitabine, Sorafenib, and Vinorelbine in high-risk patients were significantly elevated compared to low-risk patients, which indicating the high-risk patients showed a stronger drug resistance (P<0.05) (**Figures 11A, B** **)**. Similarity, patients with high-risk group were associated with increased sensitivity to Gefitinib, Vinblastine, and Sunitinib relative to low-risk patients (P<0.05) (**Figures 11A, B** **)**.

### Risk Score and TMB

**Figure 12:**
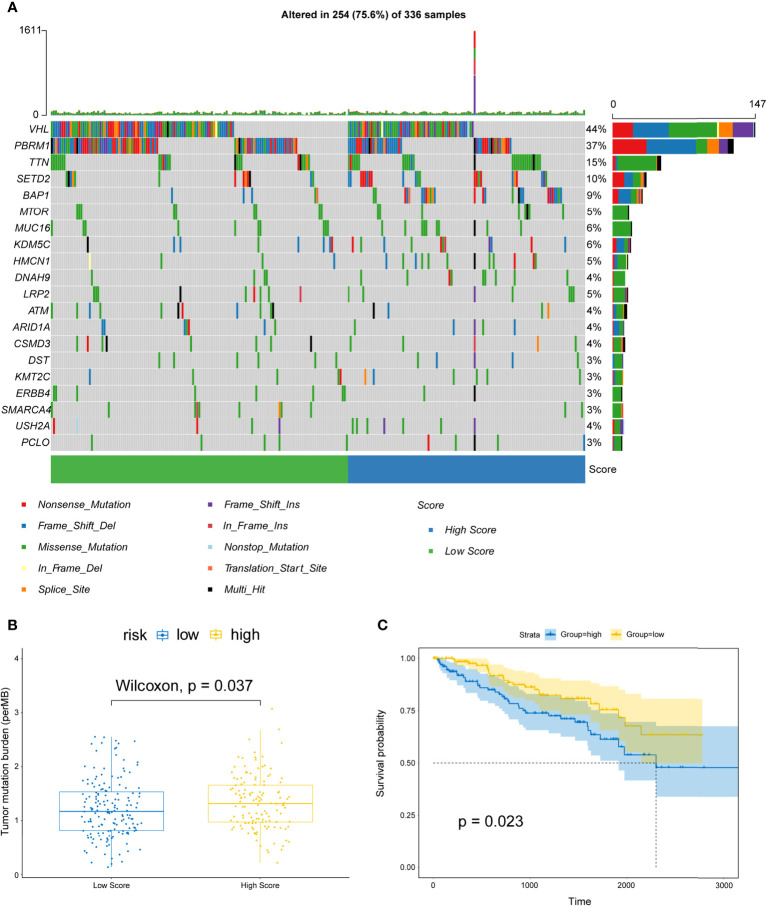
Correlations between risk scores and TMB. **(A)** OncoPrint displays the mutation profile of top 20 frequently mutated genes. Each column represents individual patients and mutated genes arranged by mutation rates. The right shows the mutation percentage, and color-coding indicates the mutation type. **(B)** Boxplot shows the difference of TMB between high- and low-risk patients. **(C)** Kaplan–Meier curves for OS divided by the high TMB group and the low TMB group.

Next, we analyzed the gene mutations of each KIRC patient. The waterfall chart showed the top 20 genes with the highest mutation frequencies: *VHL*, *PBRM1*, *SETD2*, *MTOR*, *TTN*, *MUC16*, *KDM5C*, *BAP1*, *HMCN1*, *DNAH9*, *LRP2*, *ATM*, *ARID1A*, *CSMD3*, *DST*, *KMT2C*, *ERBB4*, *SMARCA4*, *USH2A*, and *PCLO* (**Figure 12A**). Subsequently, the TMB for each sample was determined and was found to be higher in the high-risk patients (P=0.037) (**Figure 12B**) and related to shorter OS (P=0.023) than in low-risk patients (**Figure 12C**).

### Prediction of High- and Low-Risk Scores by XGBoost Algorithm

**Figure 13:**
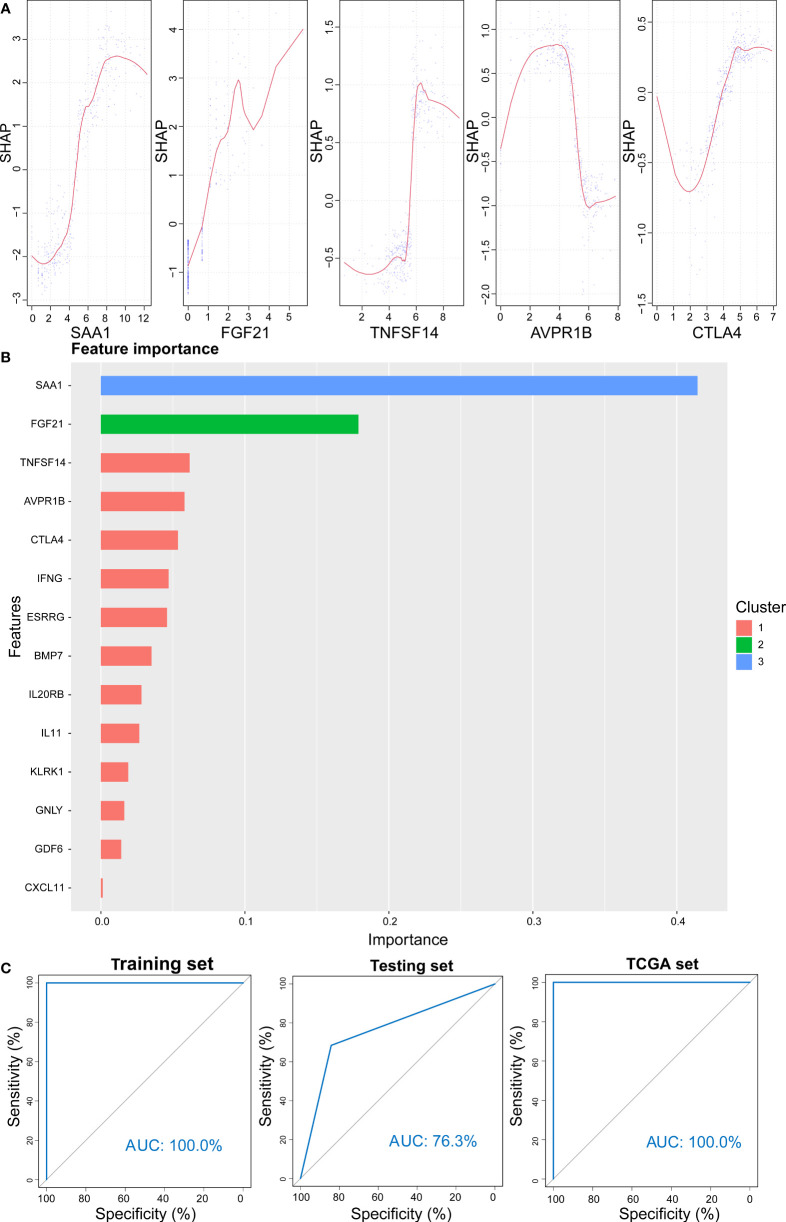
Prediction results from the XGBoost algorithm. **(A)** SHAP contribution dependency plots for the training cohort. **(B)** Importance of 14 features of the training cohort. **(C)** ROC curve for XGBoost algorithm for the prediction of high- and low-risk patients in training, testing, and entire cohorts.

XGBoost is an efficient and reliable machine learning classifier based on gradient boosting, designed to solve data science challenges accurately and rapidly in bioinformatics (62, 63). Using this approach, a classifier that could predict high- and low-risk score groups for KIRC patients based on expression levels of 14 selected genes was constructed for the training cohort. SHAP dependency plot and the importance of 14 features were visualized in **Figures 13A, B** to evaluate the contribution of each feature towards prediction. **Figure 13C** showed that the AUC of the training cohort was 100%. Then, classification model performance was assessed using the testing and entire total cohorts (**Figures S10A, B** and **Figures 13C** **)**. Taken together, the middle cutoff value might be suitable to classify KIRC patients.

### Identification of Potential Small Molecule Drugs

According to CMAP analysis, 10 small molecule drugs with highly significant correlations are listed in **Table 3**. Among these, Finasteride, Biperiden, Merbromin, Cefamandole, Fludrocortisone, and Vincamine displayed a high negative correlation and potential to improve the prognosis of RCC. Subsequently, the *SAA1* gene contributing to the model according to the feature importance was docked with these 10 compounds (**Table 4**). Next, we identified the compounds except for Orphenadrine that showed a high binding affinity against the target protein due to their binding energy <-5 kcal/mol. Moreover, the three-dimensional structure of top two high-affinity compounds combined with SAA1 is shown in **Figures S11A, B**. In SAA1-merbromin complex, due to multiple phenylene rings and active groups, merbromin forms hydrogen bonds with activity groups of amino acids, such as GLN-66, ARG-25, and TRP-53, indicating that merbromin could match well with SAA1 protein. Similarly, the SAA1-Cefamandole complex can be formed by multiple interactions, such as the cooperation of hydrogen bonding and multiple π-π stacking interactions. Hence, these two compounds were both regarded as potential SAA1 inhibitors that could improve the prognosis of RCC.

**Table 3:** The results of CMAP analysis.

rank	Cmap name	mean	n	enrichment	p	specificity
1	cetirizine	0.62	4	0.902	0.0001	0
2	finasteride	-0.385	6	-0.791	0.00016	0
3	orphenadrine	0.499	6	0.779	0.00028	0
4	biperiden	-0.516	5	-0.83	0.00034	0.0204
5	merbromin	-0.524	5	-0.807	0.00062	0.0081
6	natamycin	0.572	4	0.849	0.00074	0
7	sulfathiazole	0.515	5	0.785	0.00104	0
8	cefamandole	-0.478	4	-0.834	0.00137	0
9	fludrocortisone	-0.308	8	-0.63	0.00144	0.0704
10	vincamine	-0.539	6	-0.699	0.00171	0.0177

**Table 4:** The selected compounds of docking results.

Name	Compound Structure	Target	Binding Energy (kcal/mol)	Combination Type
merbromin	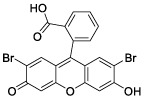	SAA1	-7.85	Hydrogen bonds, Hydrophobic interactive, π-stacking
cefamandole	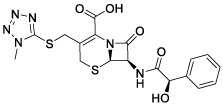	SAA1	-7.43	Hydrogen bonds, Hydrophobic interactive, π-stacking
fludrocortisone	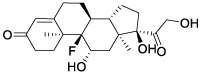	SAA1	-7.35	Hydrogen bonds, Hydrophobic interactive
cetirizine	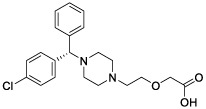	SAA1	-7.26	Hydrogen bonds, Hydrophobic interactive, π-stacking
finasteride	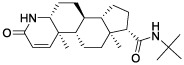	SAA1	-7.09	Hydrogen bonds, Hydrophobic interactive
vincamine	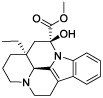	SAA1	-6.96	Hydrogen bonds, Hydrophobic interactive
sulfathiazole	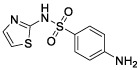	SAA1	-6.01	Hydrogen bonds, Hydrophobic interactive
biperiden	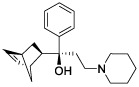	SAA1	-5.61	Hydrophobic interactive, π-stacking
natamycin	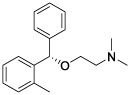	SAA1	-5.35	Hydrophobic interactive, π-stacking
orphenadrine	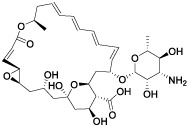	SAA1	0	0

## Discussion

Epidemiological evidence indicated that the incidence of RCC had a continually increasing trend with high mortality (64, 65). Clinical decision-making tools were effective prognostic biomarkers to predict the survival outcomes of RCC patients, rendering them a viable choice for clinicians. To date, the prognostic prediction of RCC patients relies on the TNM staging system according to the clinical practice guidelines (66). However, this system failed to taken the influence of gene level of RCC into consideration and made it not always able to predict the patients accurately. In recent years, IRGs have gradually gained attention with in-depth studies on immune-escape and immunotherapeutic mechanisms. Hence, an immune-related prognostic system is an urgent requirement for a supplementary TNM staging system.

Next, we screened for immune-associated DEGs in RCC. To minimize the potential for overfitting, 14 genes established the prognostic immune signature and were validated in TCGA through the univariate Cox proportional hazard regression and LASSO Cox analysis. Subsequently, we confirmed the independent predictive roles of this signature. Then, a personalized, predictive nomogram with a risk score was developed, which served as a predictive indicator; the signature encompassed a total of 14 IRGs. Among these, SAA1, TNFSF14, FGF21, IFNG, BMP7, and IL11 are biomarkers for predicting RCC outcomes (67–72). For example, as a member of the serum amyloid A family of apolipoproteins, SAA1 can increase the invasive capacity of tumor cells in RCC by inducing MMP-9 expression (73), which make it serve as a biomarker for the diagnosis and prognosis of advanced and metastatic renal cell carcinoma. In addition, as a member of the IL-6 family of cytokines, IL-11 exerts pleiotropic oncogenic activities may by stimulating angiogenesis and metastasis, which make it become an independent indicator of poor prognosis in RCC (71). The other IRGs, such as IL20RB, ESRRG, GDF6, were reported to be involved in the regulation of carcinogenesis (74–76) but not yet investigated in RCC. Moreover, some IRGs were also involved in TIME. For example, NKG2D receptor, KLRK1, is expressed in NK cells and activated CD8^+^ T cells, involved in innate immune responses (77). In some studies also identified GNLY as the first lymphocyte-derived alarmin protein to promote antigen-presenting cell (APC) recruitment, activation, and antigen-specific immune responses (78). CTLA-4 is a negative regulator and modulates T cell activation, and induces tolerance (79). CXCL11 is activated by IFN-γ and IFN-β and can stimulate immune cells by promoting Th1 polarization and enhancing the antitumor immunity (80). To sum up, these IRGs may affected the prognosis and treatment of RCC by influencing TIME.

Herein, some self-validation processes, including the associations between risk scores and immune cell proportions, T cell infiltrations, antitumor immunity, antitumor response, GSEA analysis, and oncogenic pathways, were conducted to identify the risk score effectiveness in characterizing the immune landscape features of RCC patients. For immunotherapeutic development, anti-PD-1, anti-CTLA-4, and anti-PD-L1 treatment have been under intensive focus in solid tumors. Nevertheless, a small number of patients respond to such treatment, and some studies (81–83) pointed out that PD-L1 and PD-1 expression levels are not reliable biomarkers to predict ICI treatment. Hence, it is necessary for clinicians to develop a reliable tool for appropriate patient selection in immunotherapy. Based on these findings, we established that the risk score is a robust immune classifier for classifying RCC patients in different subtypes. Moreover, we also demonstrated that high-score patients were more immunotherapeutically suitable compared to patients in the low-risk score group.

Targeted therapy is currently the main treatment strategy for metastatic RCC. Thus, it is necessary to identify patients with the potential to benefit from targeted therapy for RCC. Interestingly, our data showed that high-risk patients had a high sensitivity to Gefitinib, Vinblastine, and Sunitinib compared to low-risk score patients, who exhibited high sensitivity to Cisplatin, Sorafenib, Gemcitabine, and Vinorelbine. These responses could be attributed to the differences in the drug target. In addition, the TMB values of the high-risk score patients were elevated compared to those of the low-risk score patients. This finding was consistent with the concept that elevated TMB values are associated with a high probability of satisfactory immunotherapeutic outcomes (84, 85).

Nevertheless, the present study had some limitations. First, although our model exhibited precise predictive capability to predict the survival of RCC patients, multiple large external cohorts of patients with RCC are also needed to further validate. Secondly, only the median risk score was used to classify the RCC patients into high- and low-risk score subtypes. An optimal cutoff of the risk score is essential for the stratification of RCC patients. Although our model had been correlated with immune cells, the mechanism underlying poor prognosis is unclear, requiring additional experimental and theoretical studies on immune cells in RCC to further understand their functional role.

## Conclusions

Taken together, our proposed immune prognostic, predictive model could be used as a robust classifier for the prediction of survival outcomes and individual treatment guidance of adjuvant chemotherapy and anticancer immunotherapy for RCC.

## Data Availability Statement

The RNA-seq data and corresponding clinical information were observed from the TCGA (https://portal.gdc.cancer.gov/). The immune-related gene list was got from the IMMPORT website (https://www.immport.org/).

## Author Contributions

All authors participated in the design, interpretation of the studies, analysis of the data, and review of the manuscript. TF and JZ conceived and designed the whole project and wrote the manuscript. TF, DW, ZF, and ZW analyzed and visualized the data. TF, QL, PG, and XY interpreted the data and partook in the discussion. ML, YJ, and YL revised the final version of the manuscript. All authors contributed to the article and approved the submitted version.

## Funding

This study was supported by the National Science Foundation of Beijing (7172068 and 7192053).

## Conflict of Interest

The authors declare that the research was conducted in the absence of any commercial or financial relationships that could be construed as a potential conflict of interest.

## Publisher’s Note

All claims expressed in this article are solely those of the authors and do not necessarily represent those of their affiliated organizations, or those of the publisher, the editors and the reviewers. Any product that may be evaluated in this article, or claim that may be made by its manufacturer, is not guaranteed or endorsed by the publisher.

## Abbreviations

RCC, renal cell carcinoma; KIRC, kidney renal clear cell carcinoma; DEGs, differentially expressed genes; ccRCC, clear cell renal cell carcinoma; CMAP, connectivity map; ssGSEA, single sample gene set enrichment analysis; GSEA, gene set enrichment analysis; NES, normalized enrichment score; IS, immune cells; TS, tumor cells; ICB, immune checkpoint blockade; TIME, tumor immune microenvironment; TME, tumor microenvironment; TMB, tumor mutation burden; TCGA, The Cancer Genome Atlas; GEO, Gene Expression Omnibus; TCIA, the cancer immune group atlas; IRGs, immune-related genes; ImmPort, Immunology Database and Analysis Portal; DE-IRGs, differentially expressed immune-related genes; FDR, false discovery rate; LASSO, least absolute shrinkage and selection operator; ROC, receiver operating characteristic curves; C-index, concordance index; DCA, decision curve analysis; ES, ESTIMATE scores, TP, tumor purity, SS, stromal scores; IS, immune scores; CYT, cytolytic activity; PD, progressive disease; SD, stable disease; PR, partial response; CR, complete response; IPS, immunophenoscore; GDSC, Genomics of Drug Sensitivity in Cancer; PCA, principal component analysis; GO, Gene Ontology; KEGG, Kyoto Encyclopedia of Genes and Genomes; BP, biological process; CC, cellular component; MF, molecular function; PAC, antigen-presenting cell; OS, overall survival.
