# Supplementary material for: Immunogenomic Analyses of the Prognostic Predictive Model for Patients With Renal Cancer
Source: Front Immunol. 2021 Oct 12;12:762120. doi: 10.3389/fimmu.2021.762120 (PMC8546215; doi:10.3389/fimmu.2021.762120)
Supplement: Supplementary file 1 [file DataSheet_1.pdf]

Supplementary Material

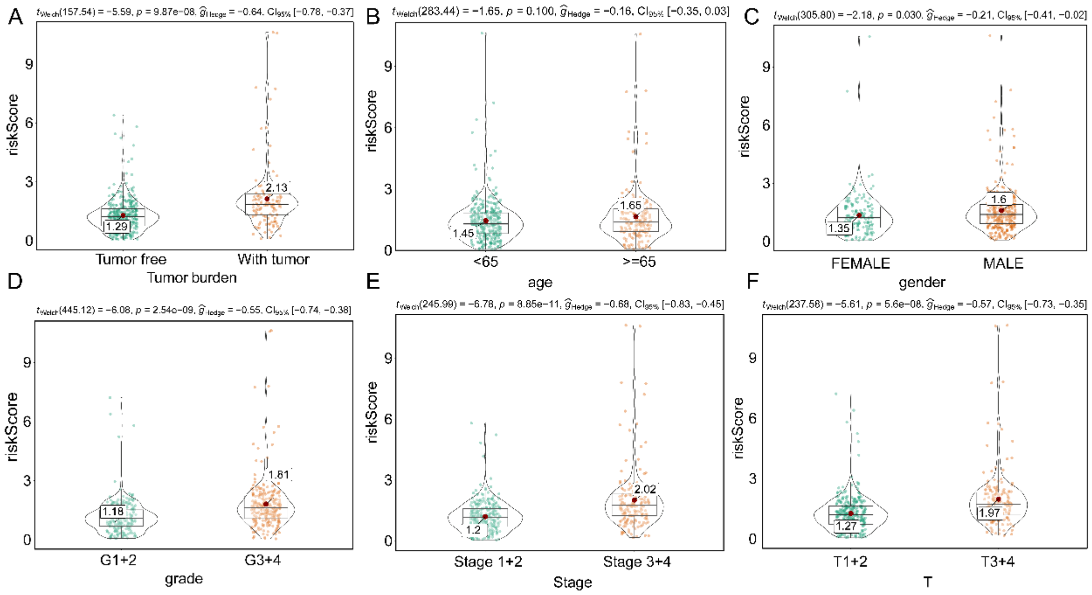

Figure S1 Violin plot of correlations between the risk score and other clinic-pathologic parameters in the TCGA cohort. (A) Tumor burden. (B) Age at diagnosis. (C) gender. (D) Grade. (E) Stage. (F) T stage.

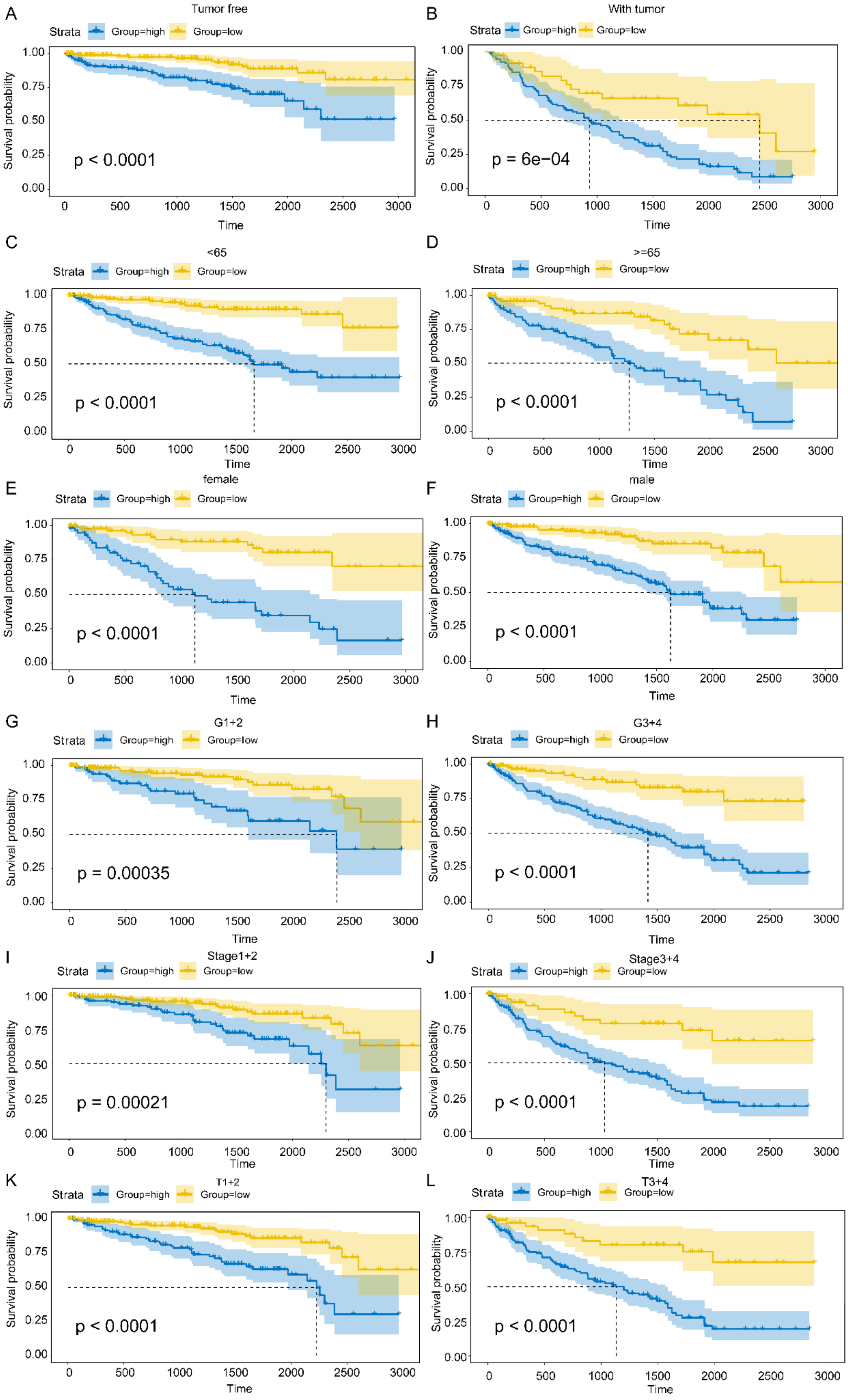
A
Tumor free
Strata
Group=high
Group=low
Survival probability
Time
p < 0.0001
B
With tumor
p = 6e−04
C
<65
p < 0.0001
D
>=65
p < 0.0001
E
female
p < 0.0001
F
male
p < 0.0001
G
G1+2
p = 0.00035
H
G3+4
p < 0.0001
I
Stage1+2
p = 0.00021
J
Stage3+4
p < 0.0001
K
T1+2
p < 0.0001
L
T3+4
p < 0.0001

Figure S2 Kaplan Meier curves between the high-risk score and the low-risk score group at different clinic-pathologic parameters in the TCGA cohort. (A) Tumor free. (B) With tumor. (C) age<65. (D) age≥65. (E) Female. (F) Male. (G) G1+2. (H) G3+4. (I) Stage1+2. (J) Stage3+4. (K) T1+2. (L) T3+4.

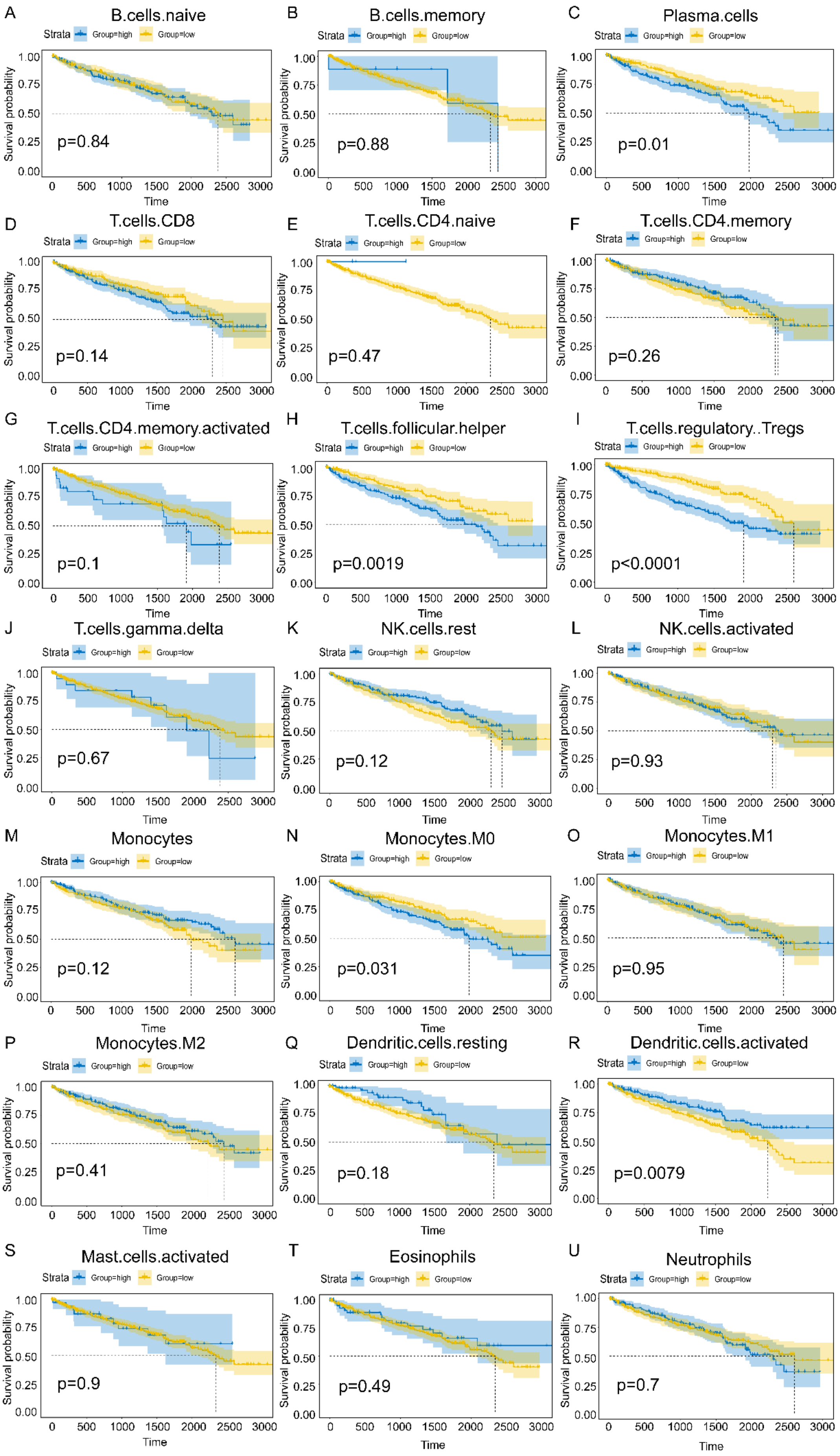
A
B.cells.naive
p=0.84
B
B.cells.memory
p=0.88
C
Plasma.cells
p=0.01
D
T.cells.CD8
p=0.14
E
T.cells.CD4.naive
p=0.47
F
T.cells.CD4.memory
p=0.26
G
T.cells.CD4.memory.activated
p=0.1
H
T.cells.follicular.helper
p=0.0019
I
T.cells.regulatory..Tregs
p<0.0001
J
T.cells.gamma.delta
p=0.67
K
NK.cells.rest
p=0.12
L
NK.cells.activated
p=0.93
M
Monocytes
p=0.12
N
Monocytes.M0
p=0.031
O
Monocytes.M1
p=0.95
P
Monocytes.M2
p=0.41
Q
Dendritic.cells.resting
p=0.18
R
Dendritic.cells.activated
p=0.0079
S
Mast.cells.activated
p=0.9
T
Eosinophils
p=0.49
U
Neutrophils
p=0.7
Strata
Group=high
Group=low
Survival probability
Time

Figure S3 Kaplan Meier curves between the high and low risk group in immune cells. The associations between overall survival and (A) B cells naïve, (B) B cells memory, (C) Plasma cells, (D) T cells CD8, (E) T cells CD4 naïve, (F) T cells CD4 memory, (G) T cells CD4 memory activated, (H) T cells follicular helper, (I) T cells regulatory Tregs, (J) T cells gamma delta, (K) NK cells rest, (L) NK cells activated, (M) Monocytes, (N) Monocytes.M0, (O) Monocytes.M1, (P) Monocytes.M2, (Q) Dendritic cells resting, (R) Dendritic cells activated, (S) Mast cells activated, (T) Eosinophils, (U) Neutrophils.

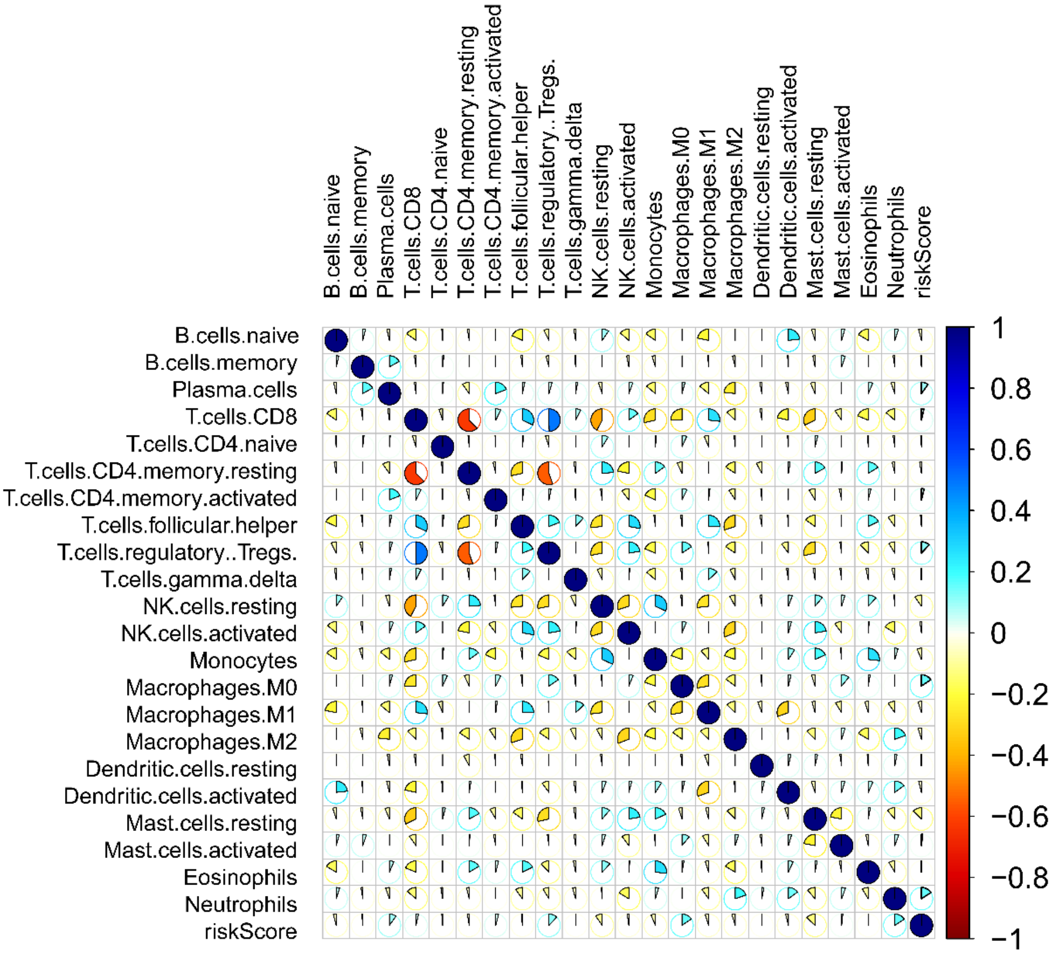

Figure S4 Correlation of the risk score with 22 immune cell proportions in the TCGA cohort.

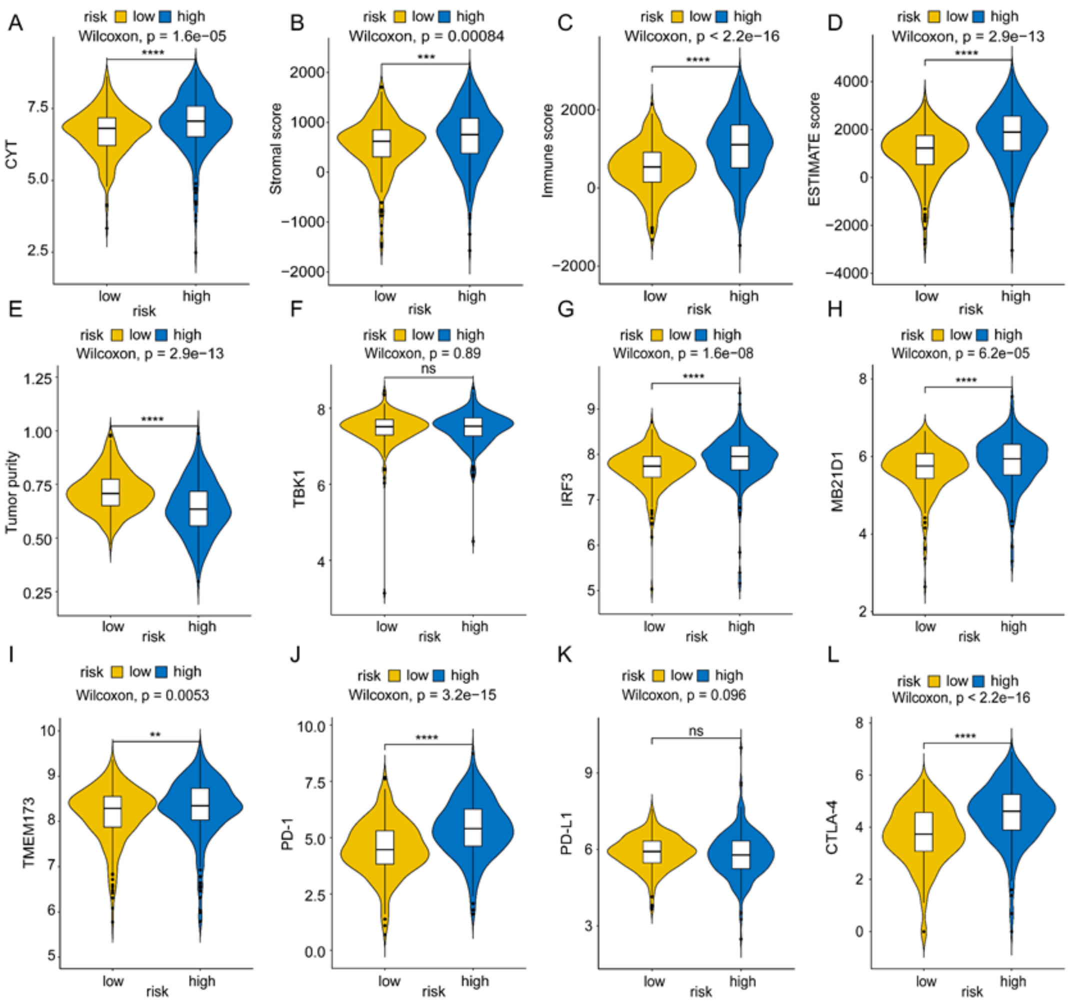

Figure S5 Violin plots for the (A) CYT, (B) Stromal score, (C) immune score, (D) ESTIMATE score, (E) Tumor purity, the expression levels of (F) TBK1, (G) IRF3, (H) MB21D1, (I) TMEM173, (J) PD-1, (K) PD-L1 and (L) CTLA-4 between two risk score subtypes. Statistical significance at the level of ns≥0.05, **<0.01, ***<0.001 and ****<0.0001.

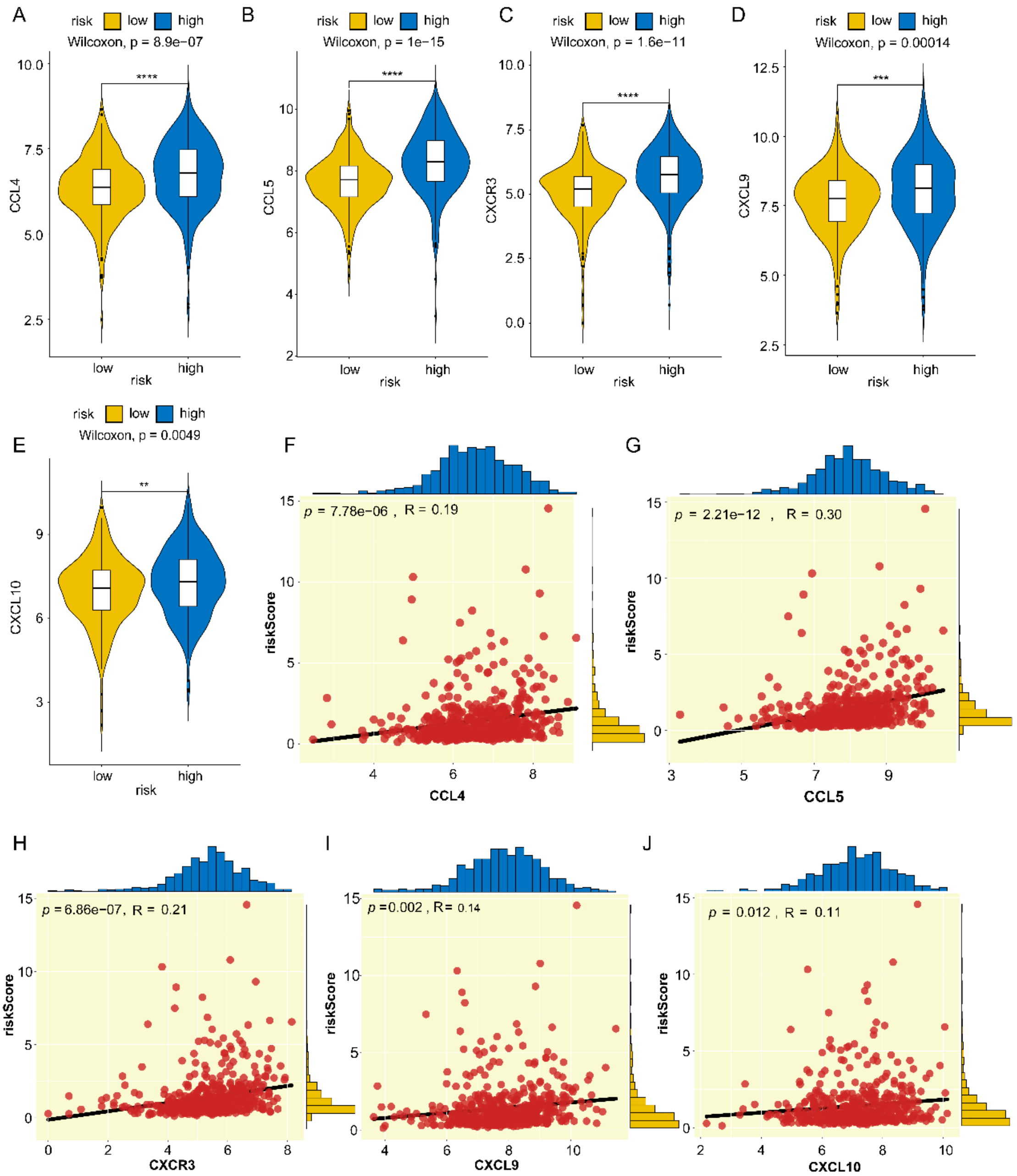

Figure S6 Correlation of the risk score with T cell and NK cell-recruiting chemokines. Violin plots of expression level of (A) CCL4, (B) CCL5, (C) CXCR3, (D) CXCL9 and (E) CXCL10 between two risk score subtypes. Statistical significance at the level of ＊＊<0.01, ＊＊＊<0.001 and ＊＊＊＊<0.0001.

Correlation of the risk score with (F) CCL4, (G) CCL5, (H) CXCR3, (I) CXCL9 and (J) CXCL10.

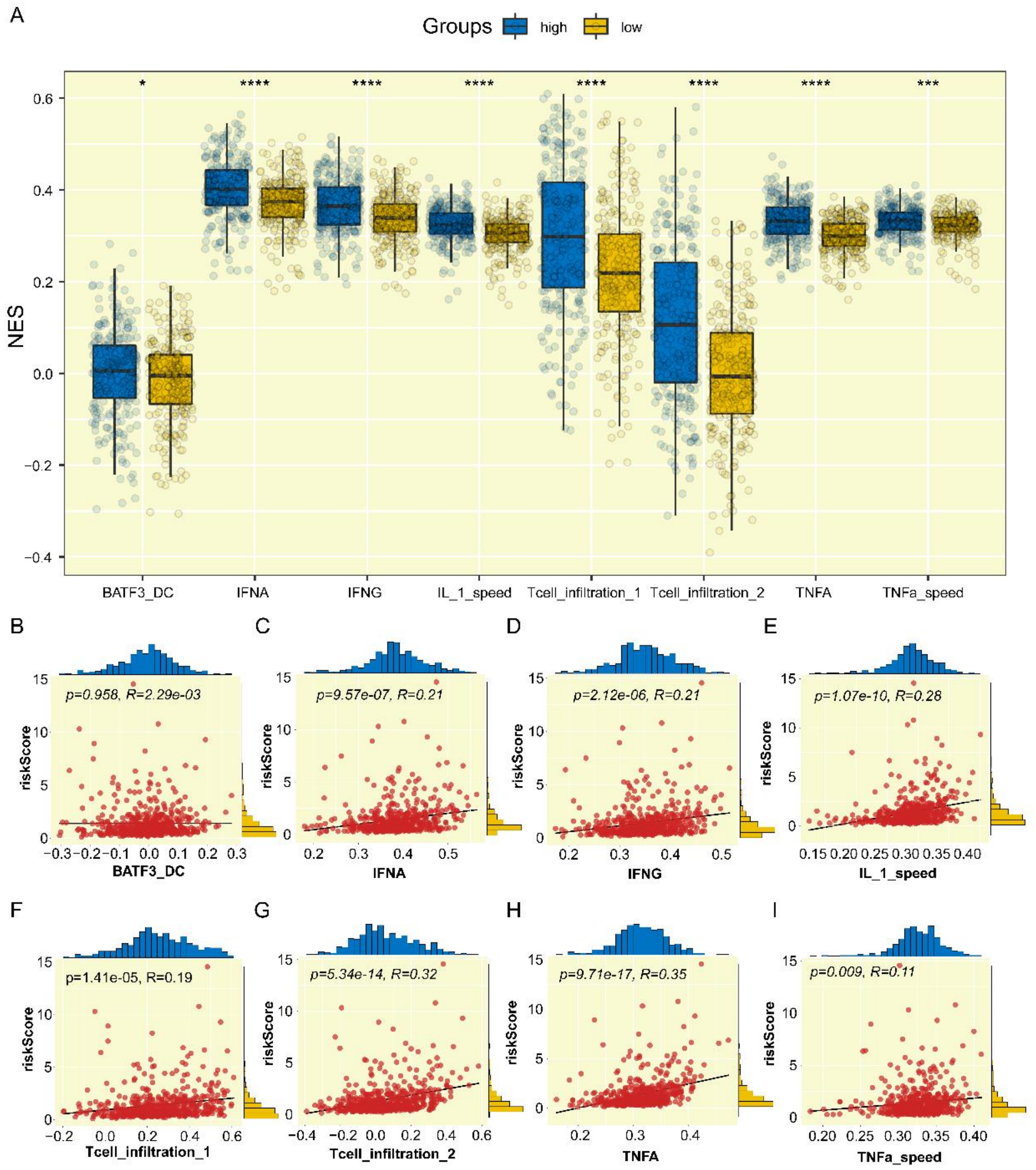

Figure S7 Correlation of the risk score with T cell infiltration and antitumor response. (A) Boxplots of the 8 indicators of T cell infiltration and antitumor response for two risk score subtypes. Statistical significance at the level of *<0.05, ***<0.001 and ****<0.0001.

Correlation of the risk score with 8 indicators (B) BATF3_DC, (C) IFNA, (D) IFNG, (E) IL_speed, (F) T cell_infiltration_1, (G) T cell_infiltration_2, (H) TNFA and (I) TNFa_speed of T cell infiltration and anti-tumor response.

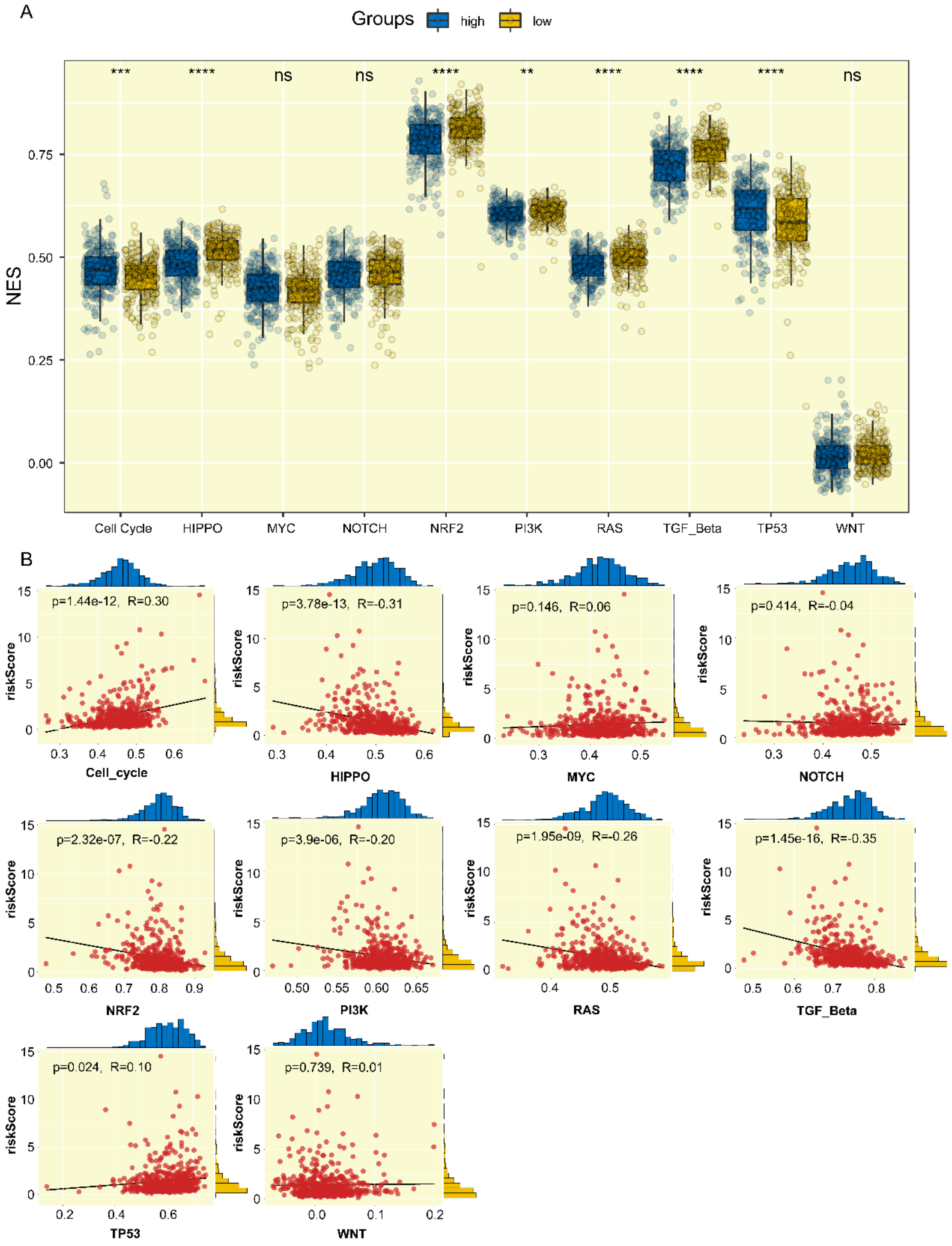

Figure S8 Correlation of the risk score with 10 oncogenic pathways.

(A) Boxplots of the 10 oncogenic pathways between two risk score subtypes. Statistical significance at the level of ns≥0.05, **<0.01, ***<0.001 and ****<0.0001. Correlation of the risk score with 10 oncogenic pathways (B) cell cycle, (C) HIPPO, (D) MYC, (E) NOTCH, (F) NRF2, (G) PI3K, (H) RAS, (I) TGF Beta, (J) TP53 and (K) WNT.

F

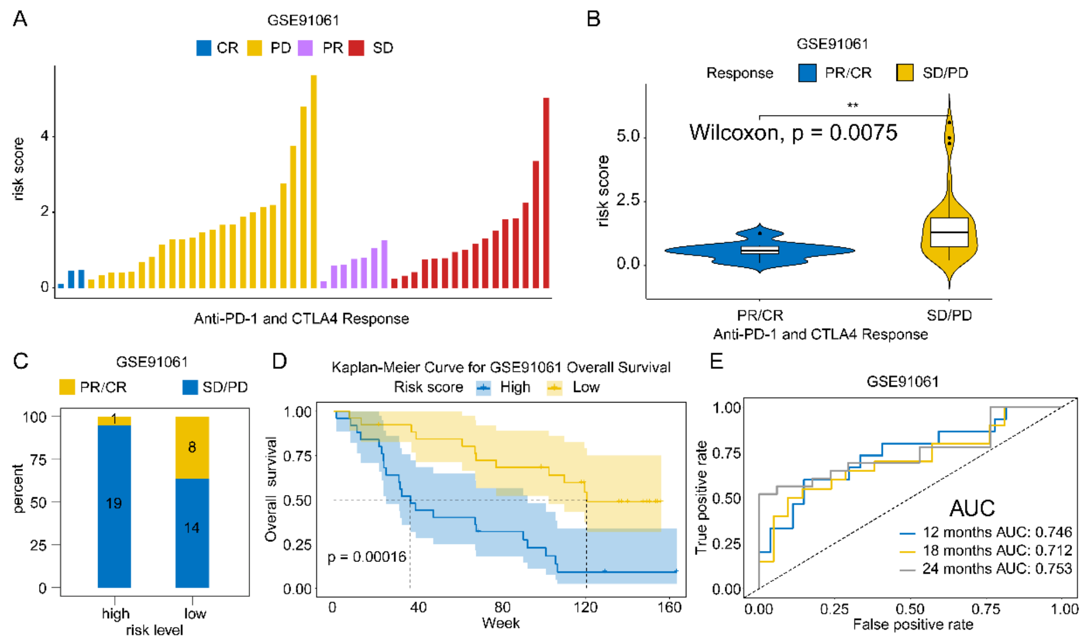

Figure S9 The therapeutic benefits of the risk score in the GSE91061 cohorts.

(A) Waterfall plot illustrating the distribution of the risk score for patients with different anti-PD-1 and CTLA4 immunotherapy responses.

(B) Violin plot illustrating the distribution of IRRS for patients with different anti-PD-1 and CTLA4 immunotherapy responses. Significance was determined by the Wilcoxon test.

(C) Bar graph illustrating the numbers of clinical responses to anti-PD-1 and CTLA4 immunotherapy in the high and low risk score subtypes (P-value=0.036, Chi square test).

(D) Kaplan-Meier curves of overall survival according to the risk score subtypes.

(E) Time-dependent ROC curves of anti-PD-1 and CTLA4 immunotherapy response prediction on the 1-, 1.5-, and 2-year survival rates.

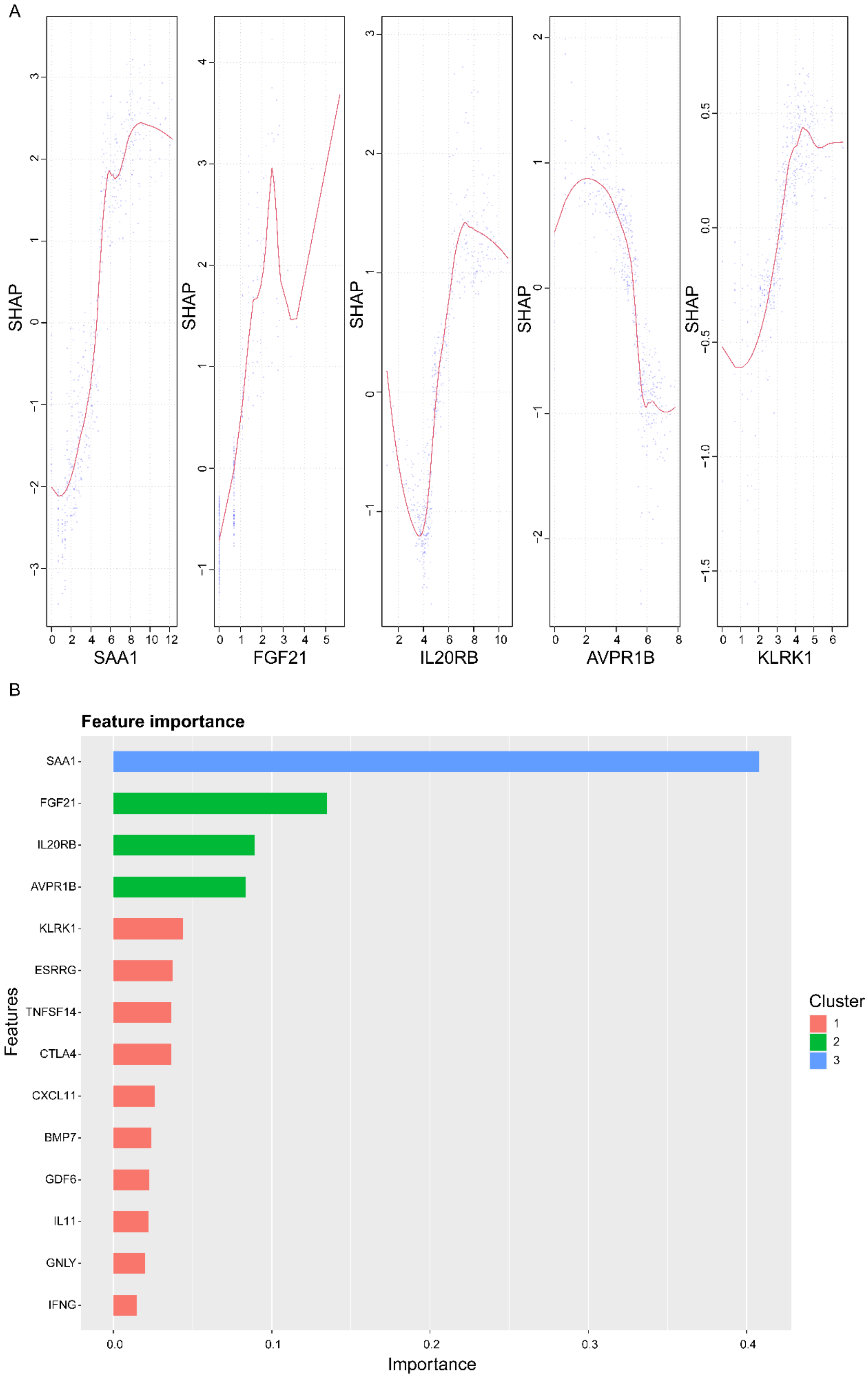

Figure S10 Prediction results of the XGBoost algorithm in the entire cohort.

(A) The SHAP contribution dependency plots for the entire cohort.
(B) The contributions of 14 importance features for the entire cohort.

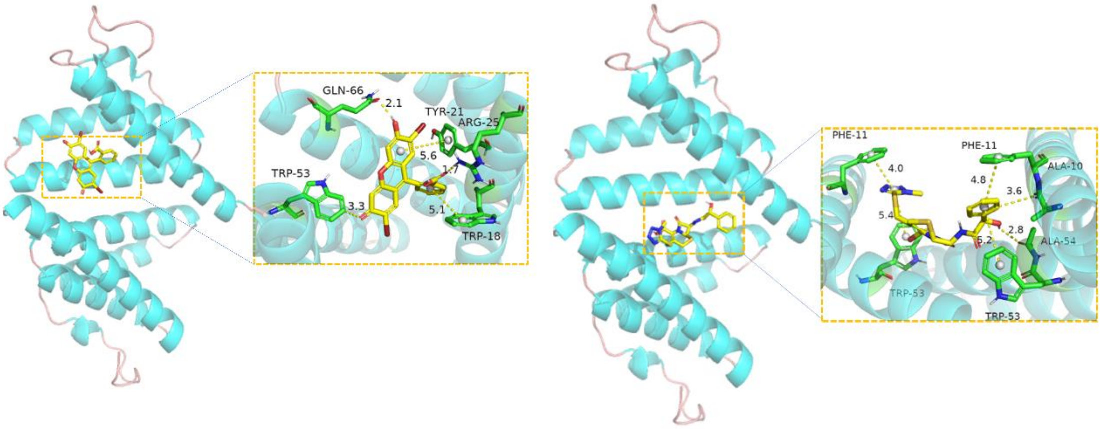

Figure S11 3D structure of SAA1-merbromin complex and SAA1-cefamandole complex based on molecular docking.
(A) The binding mode of the complex SAA1 with Merbromin.
(B) The binding mode of the complex SAA1 with Cefamandole.
